# Genome sequencing and comparative genomics reveal a repertoire of putative pathogenicity genes in chilli anthracnose fungus *Colletotrichum truncatum*

**DOI:** 10.1371/journal.pone.0183567

**Published:** 2017-08-28

**Authors:** Soumya Rao, Madhusudan R. Nandineni

**Affiliations:** 1 Laboratory of Genomics and Profiling Applications, Centre for DNA Fingerprinting and Diagnostics (CDFD), Hyderabad, Telangana, India; 2 Graduate studies, Manipal University, Manipal, Karnataka, India; 3 Laboratory of DNA Fingerprinting Services, Centre for DNA Fingerprinting and Diagnostics (CDFD), Hyderabad, Telangana, India; Fujian Agriculture and Forestry University, CHINA

## Abstract

*Colletotrichum truncatum*, a major fungal phytopathogen, causes the anthracnose disease on an economically important spice crop chilli (*Capsicum annuum*), resulting in huge economic losses in tropical and sub-tropical countries. It follows a subcuticular intramural infection strategy on chilli with a short, asymptomatic, endophytic phase, which contrasts with the intracellular hemibiotrophic lifestyle adopted by most of the *Colletotrichum* species. However, little is known about the molecular determinants and the mechanism of pathogenicity in this fungus. A high quality whole genome sequence and gene annotation based on transcriptome data of an Indian isolate of *C*. *truncatum* from chilli has been obtained. Analysis of the genome sequence revealed a rich repertoire of pathogenicity genes in *C*. *truncatum* encoding secreted proteins, effectors, plant cell wall degrading enzymes, secondary metabolism associated proteins, with potential roles in the host-specific infection strategy, placing it next only to the *Fusarium* species. The size of genome assembly, number of predicted genes and some of the functional categories were similar to other sequenced *Colletotrichum* species. The comparative genomic analyses with other species and related fungi identified some unique genes and certain highly expanded gene families of CAZymes, proteases and secondary metabolism associated genes in the genome of *C*. *truncatum*. The draft genome assembly and functional annotation of potential pathogenicity genes of *C*. *truncatum* provide an important genomic resource for understanding the biology and lifestyle of this important phytopathogen and will pave the way for designing efficient disease control regimens.

## Introduction

*Capsicum annuum* (chilli or hot pepper), a native of Mexico [1], is an important vegetable crop and an indispensable spice used in everyday cuisine throughout the world. A serious limiting factor and major constraint to chilli cultivation has been fruit rot or anthracnose disease caused by *Colletotrichum* species [2], a large genus belonging to the family Glomerellaceae (Glomerellales, Sordariomycetes) of Ascomycota. It is one of the most common and important genera of plant-pathogenic fungi [3,4], with some species being endophytic on symptomless plants [5] or saprotrophic [4]. *C*. *acutatum* and *C*. *gloeosporioides* are often associated with anthracnose of chilli [6], but *C*. *truncatum* (previously known as *C*. *capsici* [7]) is the most predominant causative species in major chilli growing countries in South-East Asia, including India [8,9]. The disease is characterized by very dark, sunken necrotic lesions with concentric rings of acervuli containing characteristic, strongly curved conidia [7]; causing both pre- and post-harvest fruit rots and reducing their quality and marketability [10]. *C*. *truncatum* is a devastating pathogen of many other tropical crops and has a wide host range including Solanaceae, Brassicaceae, Fabaceae, Malvaceae, etc. [11] and is even reported to infect humans [12]. At present, there are 11 major species complexes/clades and 23 singleton species in the genus *Colletotrichum* [11]. Thorough characterization of the species causing anthracnose and knowledge of the molecular mechanism of its progression is critical to devise strategies to efficiently control the spread of the disease.

The *Colletotrichum* species use melanized appressoria to penetrate host tissues and follow host-specific infection strategy of either intracellular hemibiotrophy or subcuticular intramural necrotrophy [13][14]. Some *Colletotrichum* species adopt both the strategies on same or different hosts; for example, members of the acutatum and gloeosporioides species complexes [14]. The initial stages of infection are similar for both groups of pathogens; conidia adhere to, and germinate on plant surfaces, produce germ tubes and form appressoria which penetrate the cuticle directly. Following penetration, in intracellular hemibiotrophic infection the primary biotrophic hyphae grow intracellularly within the cell lumen without killing the host protoplast and subsequently give rise to secondary necrotrophic hyphae, as exemplified by most of the *Colletotrichum* species like *C*. *lindemuthianum* on *Phaseolus vulgaris*, *C*. *graminicola* on maize etc. [14]. In contrast, the subcuticular intramural pathogens form an intramural network of hyphae within the walls of epidermal and cortical cells beneath the cuticle and kill the host cells prior to the rapid necrotrophic mycelial spread [11] as exemplified by *C*. *truncatum* (*C*. *capsici*) on cowpea (*Vigna unguiculata*) [13] and cotton (*Gossypium hirsutum* L.) [14]. Recent studies suggest that *C*. *truncatum* colonises chilli through subcuticular intramural necrotrophy with a brief asymptomatic, endophytic phase after initial infection and prior to necrotrophic development [15,16]. Still, it contrasts with the hemibiotrophic lifestyle that is followed by most of the *Colletotrichum* species. Despite extensive efforts to devise measures for the management and control of anthracnose, it has been difficult to control in the field because of increasing resistance in the pathogen populations to common fungicides and non-availability of cultivated varieties of *C*. *annuum* showing satisfying levels of resistance against the fungus. The lack of information on the genome content and organization has been a limiting factor in the study of this important phytopathogen and for developing strategies aimed at limiting its spread.

In the post-genomics era, an increasing number of pathogenic fungi are being sequenced, leading to the discovery of several genes and virulence factors that play key roles in host infection and disease progression. A number of secreted fungal effector molecules have been identified that suppress plant defense responses and modulate plant physiology to facilitate the host colonization [17]. These rapidly evolving effectors help fungi adapt to highly diverse lifestyles, and act as virulence factors governing the compatible interactions with the host. The whole genome sequence of many *Colletotrichum* species belonging to different species complexes, viz., *C*. *graminicol*a [18], *C*. *higginsianum* [18,19], *C*. *orbiculare* [20], *C*. *fructicola* (previously mis-identified as *C*. *gloeosporioides* Nara gc5 [21]) [20], *C*. *fiorineae* [22], *C*. *sublineola* [23], *C*. *scovillei* (previously *C*. *acutatum*, host chilli) [24], etc., are publicly available, giving an impetus to the *Colletotrichum* research. The comparative genomic analyses of important gene classes among different *Colletotrichum* species and related fungi had helped in the discovery of the core genes conserved in the genus *Colletotrichum*, and the expansion and contraction of certain gene classes that could be attributed to their specific lifestyles [18,20,25,26].

The genome of an Indian isolate of *C*. *truncatum* was sequenced, and a repertoire of putative pathogenicity genes like secretory proteases and cell wall degrading enzymes, candidate effectors, secondary metabolite (SM) biosynthesis genes, etc., were identified, which gave an insight into different aspects of its biology, lifestyle and host specificity. The comparative genomic analyses were carried out between all *Colletotrichum* species and other fungi with diverse lifestyles using both genome and proteome data that was publicly available. With the whole genome sequencing of this major member of the truncatum species complex, 9 out of 11 clades of the phylogenetic tree of the *Colletotrichum* genus presently have the genome information available, facilitating further genus-wide evolutionary and comparative studies.

## Materials and methods

### Fungal culture conditions and plant infection assay

Six *Colletotrichum capsici* cultures that were deposited in Microbial Type Culture Collection and Gene Bank (MTCC), Institute of Microbial Technology (IMTECH), Chandigarh, India with MTCC no. 2071, 3414, 8473, 9691, 10147 and 10327 were procured and were propagated on potato dextrose agar (PDA, Diffco Laboratories, France). To confirm the species identity of all the fungal cultures sourced from MTCC, internal transcribed spacer region (ITS) of nuclear ribosomal cistron and 28S nuclear ribosomal large subunit rRNA gene (LSU) were PCR amplified using the conserved universal primer pairs [27] and sequenced.

The conidial suspension was prepared by adding sterile distilled water to the 7 day old colonies on PDA and scrapping the spores off the surface of the colonies using inoculation loops. The conidia were counted using haemocytometer at different dilutions and the concentrations were adjusted to 1 x 10^6^ conidia/ml. The pathogenicity assay on *C*. *annuum* fruits was carried out through wound/drop method [2] to inoculate conidia from different fungal cultures. Koch’s postulates were tested by placing parts of lesions from inoculated chillies on PDA.

A highly virulent isolate of *C*. *truncatum* (MTCC no. 3414) was selected for all the subsequent experiments. The molecular phylogenetic analysis was carried out for the *Colletotrichum* species complexes based on multilocus alignment of five genes used in a previous study [18] (internal transcribed spacer of rRNA (ITS), chitin synthase-1 (CHS-1), histone3 (HIS3), actin (ACT) and tubulin (TUB2)) in *C*. *truncatum* (MTCC no. 3414) and 27 other *Colletotrichum* species using MEGA6 [28] by neighbour joining (NJ) method with 1000 bootstrap replicates. *Monilochaetes infuscans* was taken as an outgroup for the analysis.

Mycelia were harvested from liquid cultures grown in potato dextrose broth (PDB) and Czapeck’s medium at 28°C with shaking at 180 rpm for 3 days for DNA and RNA extractions, respectively. The fruits of chilli were surface sterilized with 1% sodium hypochlorite and inoculated with 1 x 10^6^ conidia/ml of conidial suspension using non-wound/drop method [2], covered with nylon mesh, sealed in a plastic box and incubated at room temperature for different time points. The inoculated portions of the fruits were excised, ground in liquid nitrogen and stored at -80°C until RNA extraction.

### Genome sequencing and assembly

The genomic DNA was isolated from *C*. *truncatum* (MTCC no. 3414) culture using DNeasy Plant Minikit (Qiagen, Hilden, Germany) according to the manufacturer’s instructions. Two short insert paired-end (PE, average insert size of 300 bp and 500 bp), and two long insert mate-pair (average insert size of 3000 bp and 5000 bp) genomic DNA libraries were constructed and sequenced on the Illumina HiSeq 2000 platform (2x100 bp reads) at Cofactor Genomics (St. Louis, MO, USA). The demultiplexed reads, filtered for low quality and adapter sequences, were assembled using SOAPdenovo (v 1.05) [29]. The gene space coverage was estimated with Core Eukaryotic Genes Mapping Approach (CEGMA v2.5) [30] using default parameters and Benchmarking Universal Single-Copy Orthologues (BUSCO v1) [31] using fungal gene set and *Fusarium graminearium* as a species parameter in long mode.

### RNA-sequencing (RNA-Seq) and transcriptome assembly

Non-strand-specific RNA-Seq was carried out for three *in vitro* samples, including 7 day old cultures on potato dextrose agar (PDA) supported by dialysis membrane for easy collection of fungal mass along with un-germinated conidia, 3 day old liquid cultures in Czapeck’s medium (CZ) and *in vitro* appresorial assay (APR). Three *in planta* samples which included chilli fruits inoculated with *C*. *truncatum* conidia and sampled at 0, 24 and 72 hours post inoculation (0 hpi as control, 24 hpi and 72 hpi, respectively) were also used for RNA-Seq. For *in vitro* appresorial assay, conidial suspension was placed on polystyrene petri dishes (Tarsons, India; 140 x 16 mm) in the form of droplets covering the whole surface, incubated overnight in a plastic box lined with moist tissue paper to maintain the humidity and observed under light microscope for the formation of appresoria. For RNA extraction, the petri dishes were washed with RNase-free water, appresoria were scraped off the surface using a cell scraper (Corning Inc., USA) adding Trizol reagent (Invitrogen, USA). For the rest of the samples too, Trizol method was used for RNA extraction followed by purification with RNeasy minispin columns (Qiagen, Hilden, Germany) according to the manufacturers’ instructions. The RNAs from all samples were treated with Turbo DNA-free DNase (Ambion, Texas, USA) to remove any genomic DNA contamination. RNA-seq libraries were prepared using the Illumina TruSeq 3 kit and were sequenced on Illumina HiSeq 2500 platform (2x100 bp reads) at SciGenom Labs Pvt. Ltd. (Cochin, Kerala, India). The quality check of sequenced reads (S1 Table) was performed using the FastQC suite (http://www.bioinformatics.babraham.ac.uk/projects/fastqc/).

The raw reads were quality filtered using the program Trimmomatic (v0.36) [32] and rRNA sequences were removed through SortMeRNA (v2.1) [33]. PCR duplicates were removed using PRINSEQ-lite (v0.20.4) [34] and PE reads were assembled into longer super-reads through MaSuRCA (v 3.13) [35]. All the *in vitro* and *in planta* reads were aligned to *C*. *truncatum* genome (intron sizes: 10–5000 bp) and the annotated genome of chilli (*C*. *annuum* cv. Zunla-1) [36] (intron sizes: 50–20,000), respectively using HISAT2 [37]. The *in planta* reads which did not map to chilli genome were aligned to *C*. *truncatum* genome assembly. Both genome-guided and *de novo* transcript assemblies of *C*. *truncatum* were obtained using Trinity (v2.2.0) [38] with jaccard-clip option to reduce the fusion genes. The non-redundant transcripts were retained using CD-HIT-EST [39] (95% identity threshold). Program to Assemble Spliced Alignments (PASA) [40] was used to align transcripts to genome and reconstruct the refined transcriptome. A set of likely coding regions was extracted for training *ab initio* gene prediction softwares through TransDecoder module bundled with PASA.

### Gene prediction

Genes were predicted through a combination of evidence-based (transcriptome data and homology to known proteins) and *ab initio* methods through MAKER2 (v2.31.7) [41]. Repetitive elements in the genome assembly were masked using library of all fungal repeat elements in the Repbase database (v 20140131) through RepeatMasker (v4.0.5, http://www.repeatmasker.org/) as well as the *de novo* repeat library identified using RepeatModeler (v1.0.8, http://www.repeatmasker.org/RepeatModeler.html). Genes were predicted using SNAP (v2013-02-16) [42] trained on CEGMA output, AUGUSTUS (3.0.3) [43] trained on BUSCO output, auto-trained GeneMark-ES (v4.21) [44], and GeneId (v1.4) [45]. The training set of transcripts was supplied as organism-specific ESTs to MAKER2 along with *C*. *lentis* ESTs from NCBI as alternate ESTs and the known proteins from *C*. *higginsianum* [19], *C*. *graminicola* [18], *C*. *fructicola* [20] and SwissProt database. The process was reiterated using SNAP and AUGUSTUS trained on the output of the previous run including the transcriptome assembly from PASA. The standard set of protein coding genes was compiled following the recommendations of Campbell and coworkers [46], retaining high confidence *ab initio* gene models. Some of the scaffolds were randomly inspected through Apollo genome browser [47] to check for any annotation biases. Transfer RNAs (tRNAs) were predicted using tRNAScan-SE (v1.23) [48].

### Differential gene expression analysis

RNA-Seq reads from *in vitro* and *in planta* samples were mapped onto the annotated genomes of *C*. *truncatum* and chilli, respectively, using Tophat2 [49]. The unmapped reads from *in planta* samples were retrieved through bamUtils (v 1.0.13, http://genome.sph.umich.edu/wiki/BamUtil) and mapped to *C*. *truncatum* genome through Tophat2. The differential gene expression between the samples was estimated by GFOLD (V1.1.4) [50].

### Genome synteny and phylogenetic analysis

Synteny Mapping and Analysis Program (SyMAP) v4.2 [51] was used for synteny analysis of *C*. *truncatum* genome with that of *C*. *higginsianum* with minimum size of sequences set to 10 kb and masking sequences other than the protein coding genes. For phylogenetic analysis and comparative genomics, the genomes and/or proteomes of 10 *Colletotrichum* species and 12 other related plant pathogenic fungi with diverse lifestyles (S1 Fig) were downloaded from NCBI (Table 1). A phylogenetic tree was constructed for all the fungi with *Aspergillus nidulans* as outgroup. Briefly, the tightly clustered single copy orthologues present in all the fungi were identified through Proteinortho (v5.13) [52] and were aligned to each other using MUSCLE (v3.8.31) [53]. The trimmed alignment in PHYLIP format was generated through trimAl (v1.4.rev15) [54] and concatenated to generate a supermatrix using FASconCAT (v1.0) [55]. A maximum likelihood tree with 1000 bootstraps was inferred using RAxML (v 8.2.9) [56] based on the ProtTest (v3.0) [57] estimated models (Gamma distribution of rate heterogeneity on invariant sites with JTT model and empirical amino acid frequency) and was visualized through Figtree (http://tree.bio.ed.ac.uk/software/figtree/).

**Table 1:** The list of fungal genomes used for comparative analysis with *C*. *truncatum* sequenced in the present study (in bold).

Organism	Strain	Host	Origin	Accession no.	Ref.
***C*. *truncatum***	**MTCC #3414**	***Capsicum annuum***	**India**	**NBAU00000000**	-
*C*. *graminicola*	M1.001	*Zea mays*	USA	ACOD00000000.1	[18]
*C*. *higginsianum*	IMI 349063	*Brassica rapa*	Trinidad & Tobago	LTAN00000000.1	[19]
*C*. *orbiculare*	MAFF 240422	*Cucumis sativus*	Japan	AMCV00000000.1	[20]
*C*. *fructicola* (deposited as *C*. *gloeosporioides*)	Nara gc5	*Fragaria x ananassa*	Japan	ANPB00000000.1	[20]
*C*. *fioriniae*	IMI 504882	*Fragaria x ananassa*	New Zealand	JARH00000000.1	[22]
*C*. *sublineola*	TX430BB	*Sorghum bicolor*	USA	JMSE00000000.1	[23]
*C*. *simmondsii*	CBS 122122	*Carica papaya*	Australia	JFBX00000000.1	[26]
*C*. *salicis*	CBS 607.94	*Salix* species	Netherlands	JFFI00000000.1	[26]
*C*. *nymphaeae*	IMI 504889	*Fragaria x ananassa*	Denmark	JEMN00000000.1	[26]
*C*. *incanum*	MAFF 238704	*Raphanus sativus*	Japan	LFIW00000000.1	[5]
*C*. scovillei*	1	*Capsicum annuum*	South Korea	LUXP00000000.1	[24]
*Verticillium alfalfa*	VaMs.102	*Medicago sativa*	USA	ABPE00000000.1	[58]
*V*. *dahliae*	VdLs.17	*Lactuca sativa*	USA	ABJE00000000.1	[58]
*Fusarium graminearum*	PH1	*Triticum* species	USA	AACM00000000.1	[59]
*F*. *oxysporum*	FOL 4287	*Solanum lycopersicum*	Spain	AAXH00000000.1	[59]
*F*. *verticillioides*	7600	*Zea mays*	USA	AAIM00000000.2	[59]
*Magnaporthe oryzae*^#^	70–15	*Oryza sativa*	French Guiana	AACU00000000.3	[60]
*M*. *oryzae*	P131	*Oryza sativa*	Japan	AHZT00000000.1	[61]
*M*. *oryzae*	Y34	*Oryza sativa*	China	AHZS00000000.1	[61]
*N*. *crassa*	OR74A	-	USA	AABX00000000.3	[62]
*Trichoderma reesei*	QM6a	-	Solomon Islands	AAIL00000000.2	[63]
*Botrytis cinerea*	B05.10	Unknown	Germany	ASM83294v1	[64]
*Sclerotinia sclerotiorum*	1980	*Phaseolus vulgaris*	USA	AAGT00000000.1	[64]

*Gene annotations were not available for further analyses at the time of writing this manuscript.

^#^ The strain used for phylogenetic analysis.

### Functional annotation

Putative functions were assigned to the predicted proteins that had at least one homologue identified through BLASTP (v2.2.26) against the SwissProt database (E-value threshold 1e-05) and/or a known protein domain identified through InterProScan 5 (v5.20–59.0) [65]. Secretome was predicted using a battery of tools; Signal-P (v4.1) [66], Phobius [67] and WoLF PSORT (v0.2) [68] to identify signal peptides or extracellular localization, and TMHMM (v2.0) [69], PS-SCAN (ftp://ftp.expasy.org/databases/prosite/ps_scan/ps_scan.pl) and PredGPI (http://gpcr.biocomp.unibo.it/predgpi/pred.htm) to exclude sequences with transmembrane domains, ER-retention signal and GPI anchors, respectively. The secretory proteins with nuclear localization signal were identified using NLStradamus (v1.8) [70], while putative effector proteins were predicted through EffectorP (v1.0) [71]. Carbohydrate Active enZymes (CAZymes) were identified by HMMER (v3.1b1, http://hmmer.janelia.org/) scan against the family-specific HMM profiles of dbCAN release 2.0 [72]. Putative proteases and protease inhibitors were predicted through online batch BLAST of proteins against the MEROPS database [73], excluding the sequences with incomplete domains and mutated active sites or metal ligands. Secondary Metabolite Unique Regions Finder (SMURF) [74] was used to identify potential secondary metabolite (SM) gene clusters. In addition to SMURF, another online tool, antibiotics and Secondary Metabolite Analysis SHell (antiSMASH fungal version 4.0.0rc1) [75] was also used with default parameters for the prediction of SM gene clusters in *C*. *truncatum*. The putative pathogenicity genes, transporters and cytochrome P450 genes were identified by retaining the best hits through BLASTP (E-value threshold 1e-10) against pathogen-host interactions database (PHI-base) [76], transporter classification database (TCDB) [77] and cytochrome P450 database [78], respectively (all accessed in October 2016). For comparative genomics, the same pipeline was run on the predicted proteomes of other *Colletotrichum* species and related ascomycete fungi downloaded from NCBI.

## Results

### Fungal culture authentication

The six *C*. *capsici* (*C*. *truncatum*) cultures procured from MTCC were authenticated by sequencing internal transcribed spacer region of nuclear rRNA (ITS), a universal DNA barcode marker, and 28S nuclear ribosomal large subunit rRNA gene (LSU), which sometimes discriminates species on its own or when combined with ITS. Conserved universal primer pairs for ITS and LSU were used to PCR amplify and sequence these genes. BLAST analysis against the NCBI non-redundant database showed 100% homology with *C*. *truncatum*. Pathogenicity and virulence of the cultures showing conidiation were established by inoculating chilli fruits and proving Koch’s postulates. A highly virulent fungal strain (MTCC no. 3414), isolated from chilli in Puducherry, India was selected for the subsequent experiments and genome sequencing. The molecular phylogenetic analysis based on five conserved genes of *Colletotrichum* species from different clades showed that *C*. *truncatum* (MTCC no. 3414), together with the other isolate of *C*. *truncatum* that infects *Phaseolus lunatus*, forms the truncatum clade, which diverged from the gloeosporioides clade (S2 Fig). The position of *C*. *truncatum* in the cladogram confirmed its identity in the *Colletotrichum* species complexes.

### Genome and transcriptome assembly of *C*. *truncatum*

The genome of an Indian isolate of *C*. *truncatum* was sequenced on Illumina HiSeq 2000 platform, generating a high quality draft genome assembly consisting of 6,193 contigs arranged in 81 scaffolds with a total size of 55.3 Mb and N50 scaffold length of 1.66 Mb (Table 2). The estimated genome size and G-C content were comparable to most of the sequenced species of *Colletotrichum* genus (S1 Fig). A single scaffold (scaffold_70_2.0) of ~900 bp was excluded from assembly since it had adapter contamination. None of the sequences in the *C*. *truncatum* assembly showed any homology to complete mitochondrial genome sequences of *C*. *acutatum* and *C*. *graminicola*. The completeness of the genome assembly of *C*. *truncatum* was assessed using CEGMA that showed 235 complete genes out of 248 orthologues of core eukaryotic genes (CEGs) conserved in all eukaryotes in the assembled genome (94.76% coverage). However, all the partial and missing CEGs were identified through TBLASTN comparison of orthologues from other *Colletotrichum* species to *C*. *truncatum* genome (E-value < 1e-10, >70% identity and query coverage) representing 100% coverage of the gene space. Another tool BUSCO, based on 1,438 single-copy genes present in at least 90% of the fungi, reported 99% coverage of complete BUSCOs with 1,325 single-copy orthologues detected in the *C*. *truncatum* genome assembly.

**Table 2:** Assembly statistics of the *C*. *truncatum* genome assembly.

Total number of contigs	6,193
Total number of scaffolds	81
Total length (bp)	55,368,957
Mean length (bp)	683,567
Max length (bp)	3,859,631
Min length (bp)	906
Largest 10 Total (bp)	26,251,402
Scaffold N50 (bp)	1,667,354
Number of Gaps	6,793
Total Gap Length (bp)	2,630,941
Mean Gap Length (bp)	387
Percent N's	4.75%
GC content	49.61%
Repetitive DNA (homology based/ *de novo* method, S2 and S3 Tables)	0.44–3%
tRNA	257
Refined transcripts	32,566
Predicted protein coding genes	13,724
Coding region in genome	51%
Genes with SwissProt homologues	66%
Genes with IPR/Pfam annotations	74%
Genes with GO annotations	46%
Genes with AED scores between 0–0.25	82%

RNA-Seq was carried out with three *in vitro* samples of *C*. *truncatum* and three *in planta* samples of inoculated chillies at different time points giving 13.7–39.5 million processed PE reads (S1 Table). ~90% of *in vitro* processed reads (super-reads, PEs and singletons) mapped to the *C*. *truncatum* genome, while >88% of reads from each *in planta* sample mapped to the chilli genome. The reads from *in planta* samples which failed to map on the chilli genome were aligned to the *C*. *truncatum* genome. Few reads (0.0048%) from 0 hpi mapped to the fungal genome, while a total of 3–6% reads from 24 and 72 hpi mapped to the fungal genome. The processed reads from three *in vitro* samples and the *in planta* reads mapping to *C*. *truncatum* genome were used for *de novo* transcriptome assembly using Trinity and comprising of 57,383 transcripts, while genome-guided assembly resulted in 38,425 transcripts. 94,309 non-redundant transcripts from both the assemblies were retained. The protein coding ORFs were predicted by aligning these transcripts to *C*. *truncatum* genome through PASA, generating a refined transcriptome with 32,566 sequences. 25,208 longest full length ORFs identified by TransDecoder module of PASA were taken as training set for *ab initio* gene model prediction.

### Genome annotation

The genome annotation was performed using MAKER2 pipeline by integrating the species-specific RNA-Seq evidence, protein homology from SwissProt database and other *Colletotrichum* species, and *ab initio* gene predictions to get consensus gene models. A total of 13,724 protein coding genes were predicted in *C*. *truncatum* assembly. Annotation Edit Distance (AED), a quantitative measure that shows the high congruency of each annotated transcript with its supporting evidence if it is close to 0, was used to assess the annotated genes [46,79]. 11,315 genes had AED scores between 0 and 0.25, while 10,807 genes had AED of <0.2. In contrast, only 364 genes had AED between 0.75 and 1. Some of the gene models were randomly inspected for any annotation bias. 9,074 predicted proteins had known homologues in the SwissProt database. A functional annotation could be assigned through InterProScan to 1,522 out of the 4,650 proteins which had no homologues in SwissProt database. 10,189 proteins with a recognizable InterPro (IPR) and/or Pfam domain were identified in *C*. *truncatum* with gene ontology (GO) terms assigned to 6,385 predicted proteins (S4 Table). Interestingly, only 65% of genes with AED scores of <0.25 had a Pfam domain, whereas ~80% of genes with AED scores of >0.75 had a known domain. 31 of the 37 Pfam domains associated with fungal transcription factors (TFs) [80] were observed in 493 proteins in *C*. *truncatum* proteome. The most abundant domains, Zn(2)-Cys(6) binuclear cluster domains (PF00172) and fungal specific transcription factor domain (PF04082) were found in 169 and 107 proteins, respectively, whereas 125 proteins shared both the domains (S4 Table).

### Genome synteny

**Fig 1:**
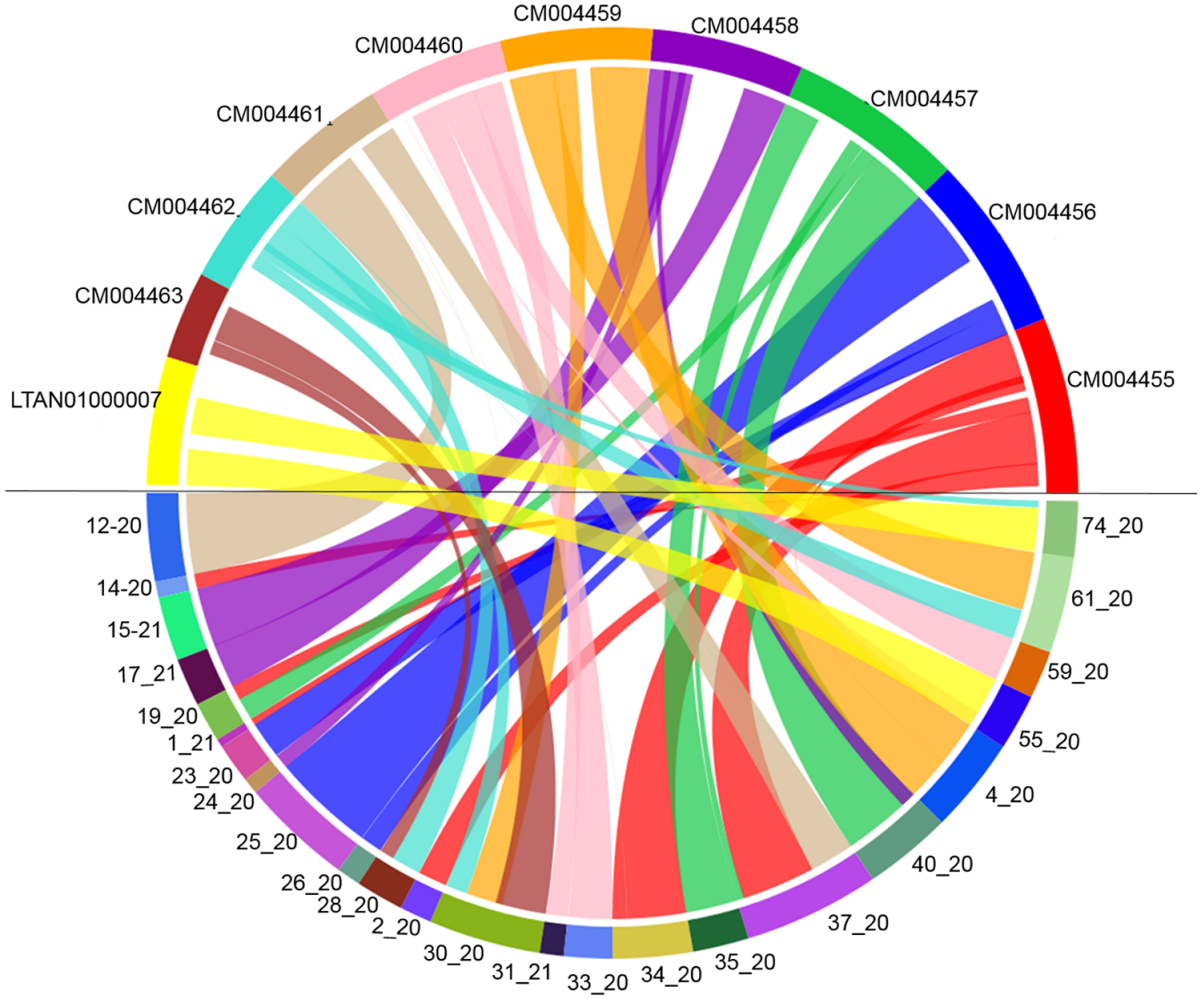
The synteny plot between *C*. *higginsianum* and *C*. *truncatum* genomes obtained using SyMAP. The circular plot shows some of the syntenic blocks between the scaffolds of *C*. *truncatum* in lower half of the circle mapping to the reference chromosomes of *C*. *higginsianum* in upper half joined with the lines of same colour as the reference.

The comparison of *C*. *truncatum* genome assembly and gene annotation with one of the well annotated species, *C*. *higginsianum* revealed 65 syntenic blocks with conserved gene order among the two genomes (Fig 1). 44 scaffolds of *C*. *truncatum* could be aligned with 9 chromosomes and a contig of *C*. *higginsianum* with 9,255 syntenic hits between the two species. The size of conserved syntenic blocks ranged from 14 kb to 3.1 Mb with 18 blocks of sizes above 1 Mb. Most of the syntenic regions were inverted in *C*. *truncatum* and fragmented synteny was observed in many regions. The synteny plots showed many translocations, like 1.7 Mb of proximal and 0.9 Mb of distal regions of scaffold 61_20 of *C*. *truncatum* were in synteny with regions on chromosome 9 (CM004462) and chromosome 5 (CM004459) of *C*. *higginsianum*, respectively (S5 Table).

### Orthologue analysis and phylogenetic relationship of *C*. *truncatum* with other fungi

**Fig 2:**
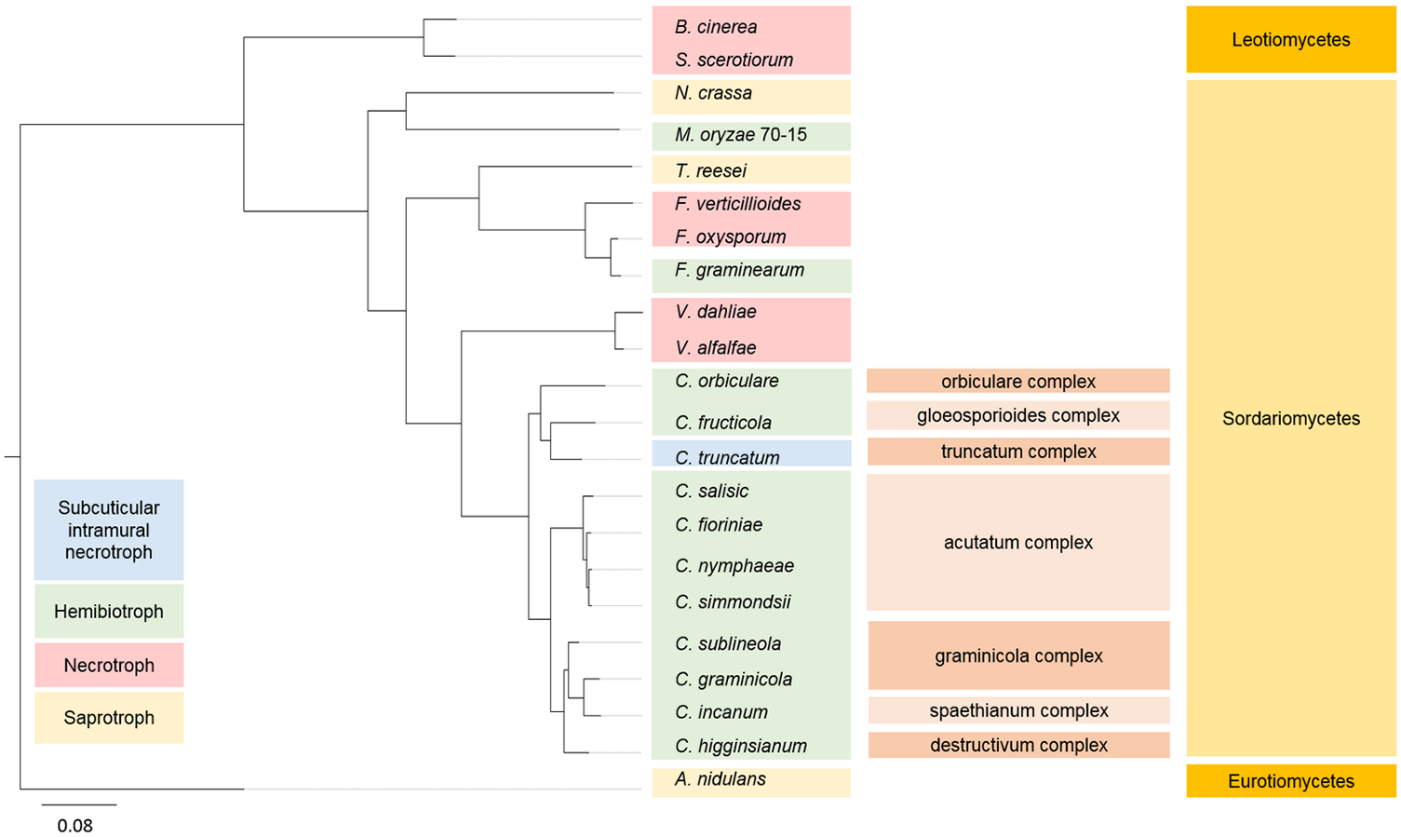
A maximum likelihood tree of *Colletotrichum* species and other fungi with diverse lifestyles. Bootstrap support values (1000 replicates) of 100% were obtained at the nodes. *A*. *nidulans* was taken as an outgroup for the analysis. The *Colletotrichum* species complexes and the families are shown in parallel. *C*. *truncatum*, *C*. *gloeosporioides* and *C*. *orbiculare* form a separate clade in the *Colletotrichum* genus suggesting their origin from a common ancestor.

A phylogenetic tree was constructed based on the concatenated alignment of 515 highly conserved, single-copy orthologues of *C*. *truncatum* with *Colletotrichum* species and other ascomycete fungi with diverse lifestyles (Table 1). The inferred phylogeny showed that the genus *Colletotrichum* was most closely related to the *Verticillium* species and the two genera diverged from *Fusarium*, while *C*. *truncatum* and *C*. *fructicola* diverged from *C*. *orbiculare* (Fig 2). Interestingly, *C*. *truncatum* had more homologues in *Fusarium* species, specifically in *F*. *oxysporum* than in any other fungus outside the genus *Colletotrichum*. Orthologue analysis of *Colletotrichum* genus using Proteinortho showed that 88.8% of the *C*. *truncatum* genes (12,186) had an orthologue in at least one of the ten *Colletotrichum* species. There were 6,081 orthogroups consisting of proteins from all the *Colletotrichum* species, out of which, 6,010 contained single-copy orthologues. 1,409 genes of *C*. *truncatum* that did not cluster in orthogroups were subjected to BLASTP against the proteomes of other *Colletotrichum* species. The gene annotation and proteome of *C*. *scovillei*, which follows a subcuticular necrotrophic lifestyle on chilli, were not in the public domain at the time of writing this manuscript. Hence, only orthologue analysis was carried out through genome-wide comparison of *C*. *truncatum* genes. TBLASTX analysis showed that 12,792 of the total *C*. *truncatum* genes had homology in *C*. *scovillei* genome, of which 26 genes had a homologue only in *C*. *scovillei* among all *Colletotrichum* species. 377 genes had no homologues in any other species used in this study or SwissProt database, and hence represented the species-specific genes that are unique to *C*. *truncatum*.

### Secretome and effector prediction

**Fig 3:**
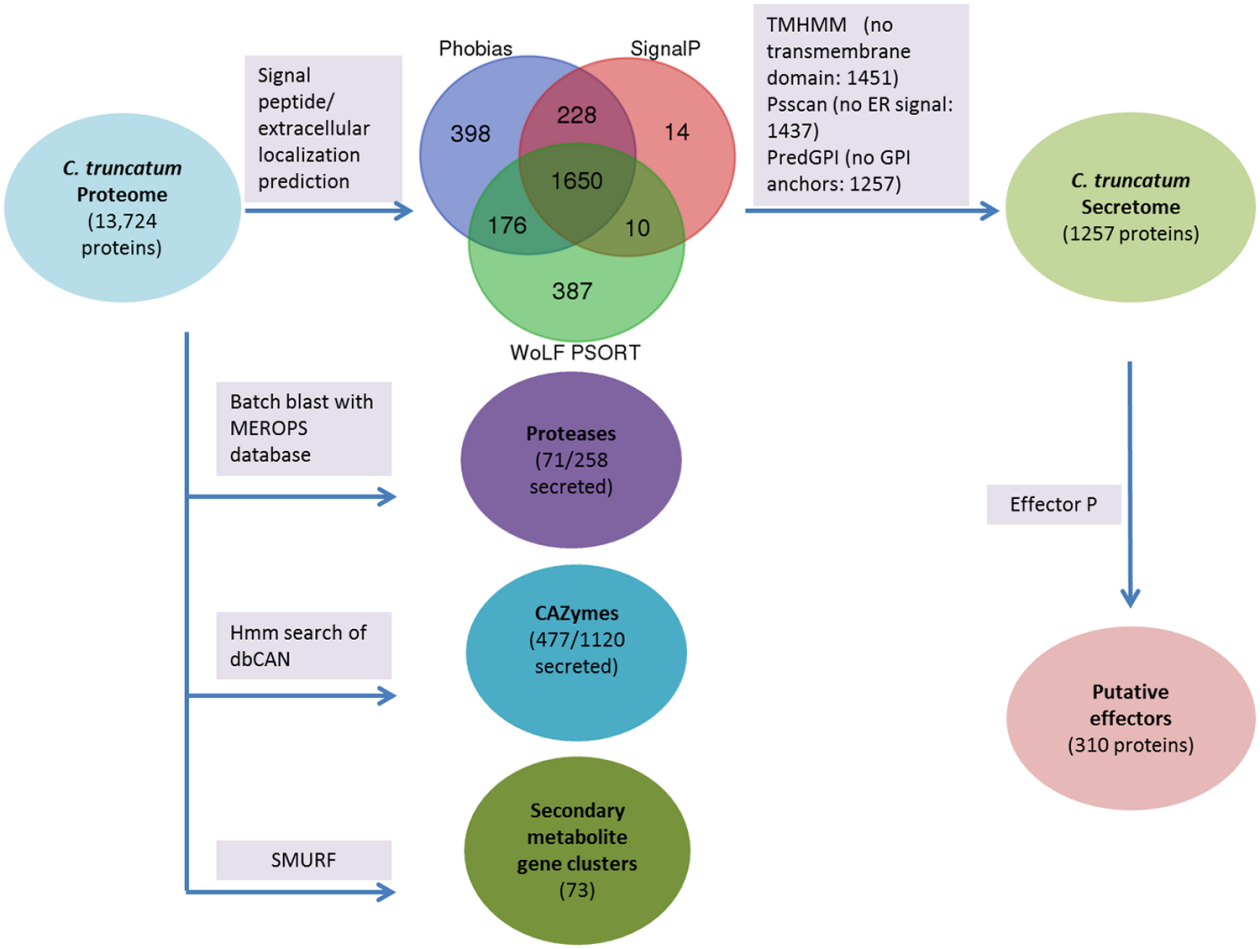
Work flow for functional annotation of some of the gene categories relevant to pathogenicity. A stringent pipeline of tools was used to predict 1257 proteins that are highly likely to be secreted and other important categories like carbohydrate active enzymes (CAZymes), proteases and secondary metabolism (SM) gene clusters. 310 of the secreted proteins were predicted to be putative effectors, 477 were secreted CAZymes and 71 were secreted proteases. Secondary metabolite backbone genes were not detected in the secretome.

The secretome of a pathogen includes extracellular secreted proteins that are deployed to the host–pathogen interface during infection and includes important virulence factors such as effector proteins for manipulation of host cell dynamics and cell wall degrading enzymes. A highly stringent pipeline for secretome prediction in fungi, as described in FunSecKB2 [81] (Fig 3), was implemented to predict a refined secretome in *C*. *truncatum* (1,257 proteins) that represented 9.16% of the total proteome. The analysis of known domains in the secretome showed that it is rich in the plant cell wall degrading enzymes like proteases and CAZymes. 793 secretory proteins had Pfam annotations, most of which were associated with subtilisin-like serine proteases (40), oxidoreductases with FAD domain (28) and carbohydrate-binding modules (28). 310 putative effector proteins were predicted by EffectorP in the secretome. The homologues of some of the known effectors from other fungi, which were detected in other *Colletotrichum* species as well, were also identified in *C*. *truncatum*; like *ChEC6* of *C*. *higginsianum*, an effector expressed specifically during appresorial penetration [82], necrosis and ethylene-inducing peptide 1 (Nep1)-like proteins (NLPs), etc. (S6 Table). 59 proteins in the predicted secretome contained nuclear localization signal, 6 of which were specific to *C*. *truncatum* with no homology in *Colletrotrichum* species, while one had a homologue in C. *scovillei* only (CTRU_006472) and 15 of these were putative effectors, which may modulate the activity of host genes by localization into the host nucleus during infection. Most of the putative effectors in *C*. *truncatum* and other fungi were cysteine-rich, small, secreted proteins (SSPs, <300 amino acids, >3% cysteine) (S3 and S4 Figs) and lacked homology to any known protein in the SwissProt database, which are the hallmarks of effectors. The sizes of all the putative effectors in *C*. *truncatum* were below 358 amino acids (aa) except for *CTRU_010949* (508 aa). 254 effectors of *C*. *truncatum* had orthologues in other fungi used in phylogenetic analysis, while 2 effectors had homologues only in *C*. *scovillei*. 75 of the 310 putative effectors had Pfam annotations, some of which were associated with enzymatic domains like pectin lyases (7), cutinase (6) and GDSL-like lipase/acylhydrolase family (5). Hence, 219 secreted proteins lacking homologues in the SwissProt database with no known function or Pfam domains were considered as candidate effectors for further functional studies (S4 Table). 34 secreted proteins of *C*. *truncatum* were specific to this species, of which 23 proteins lacking homology to any of the protein in SwissProt database were considered as species-specific effectors (S7 Table).

### CAZymes

**Fig 4:**
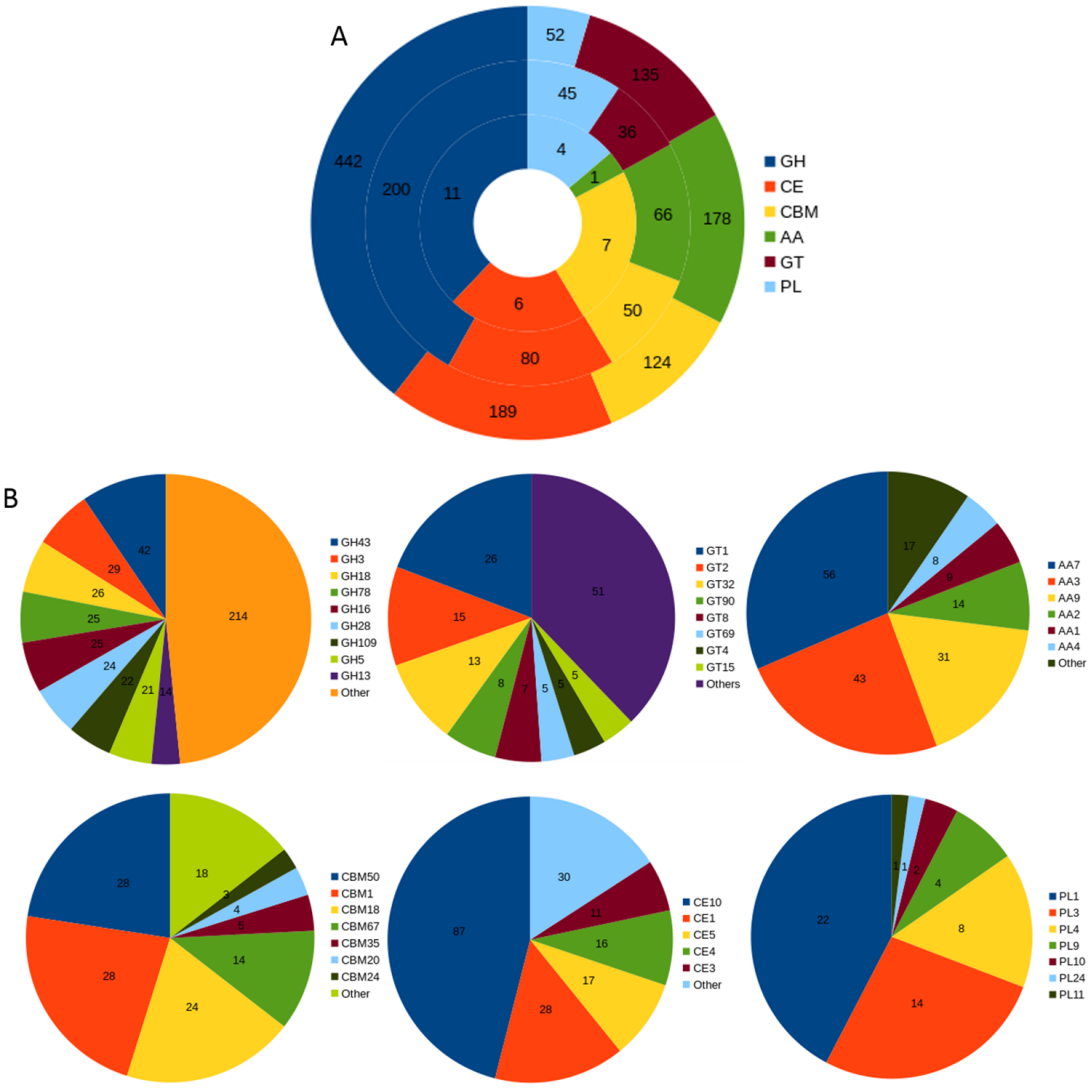
Analysis of different CAZyme families in *C*. *truncatum*. (A) The total number and category of CAZymes in predicted proteome (outer circle), secretome (middle circle) and putative effectors (inner circle) are shown. (B) The fraction of genes of major families of all the CAZyme classes, viz., glycoside hydrolases (GHs), glycosyltransferase (GTs), auxillary activities (AAs), carbohydrate-binding modules (CBMs), carbohydrate esterases (CEs) and polysaccharide lyases (PLs) are shown.

The carbohydrate metabolizing enzymes play an important role in the degradation of fungal and plant cell wall components (chitin, cellulose, hemicellulose, pectin, etc.) and utilization of plant polysaccharides during the host colonization by pathogenic fungi [83–85]. The *C*. *truncatum* genome had 1,036 genes encoding 147 different CAZyme families (Fig 4). 449 of *C*. *truncatum* genes associated with 88 CAZyme families were predicted to be secreted. The important classes of CAZymes like glycoside hydrolases (GH), carbohydrate esterases (CE), polysaccharide lyases (PL), auxillary activities (AA) and carbohydrate-binding modules (CBMs, lack catalytic activities but are included due to their association with catalytic modules) in *C*. *truncatum* were compared to other *Colletotrichum* species and other fungi. 84 CAZyme families were common in all the fungal species analysed (S8 Table), with choline esterases (CE10) representing the most abundant family (though most of the members of this family have non-carbohydrate substrates) followed by chitooligosaccharide oxidase (AA7). Cellobiose dehydrogenases (AA3, flavoproteins containing a flavin-adenine dinucleotide (FAD)-binding domain) was the third largest CAZyme family in all *Colletotrichum* species as well as in Leotiomycetes (*Botrytis* and *Sclerotinia*). The family AA9 with cellulase activity, which was previously designated as GH60, was also highly expanded in *Colletotrichum* species. The family GH43, with both hemicellulase and pectinase activities, was expanded in *C*. *truncatum* (42), *C*. *fructicola* (40) and acutatum species complex (35–39) as compared to other species in the genus (16–30). Species-specific expansion of one glycosyl transferase domain GT1 in *C*. *truncatum* (26) was also observed among all the fungi analysed.

**Fig 5:**
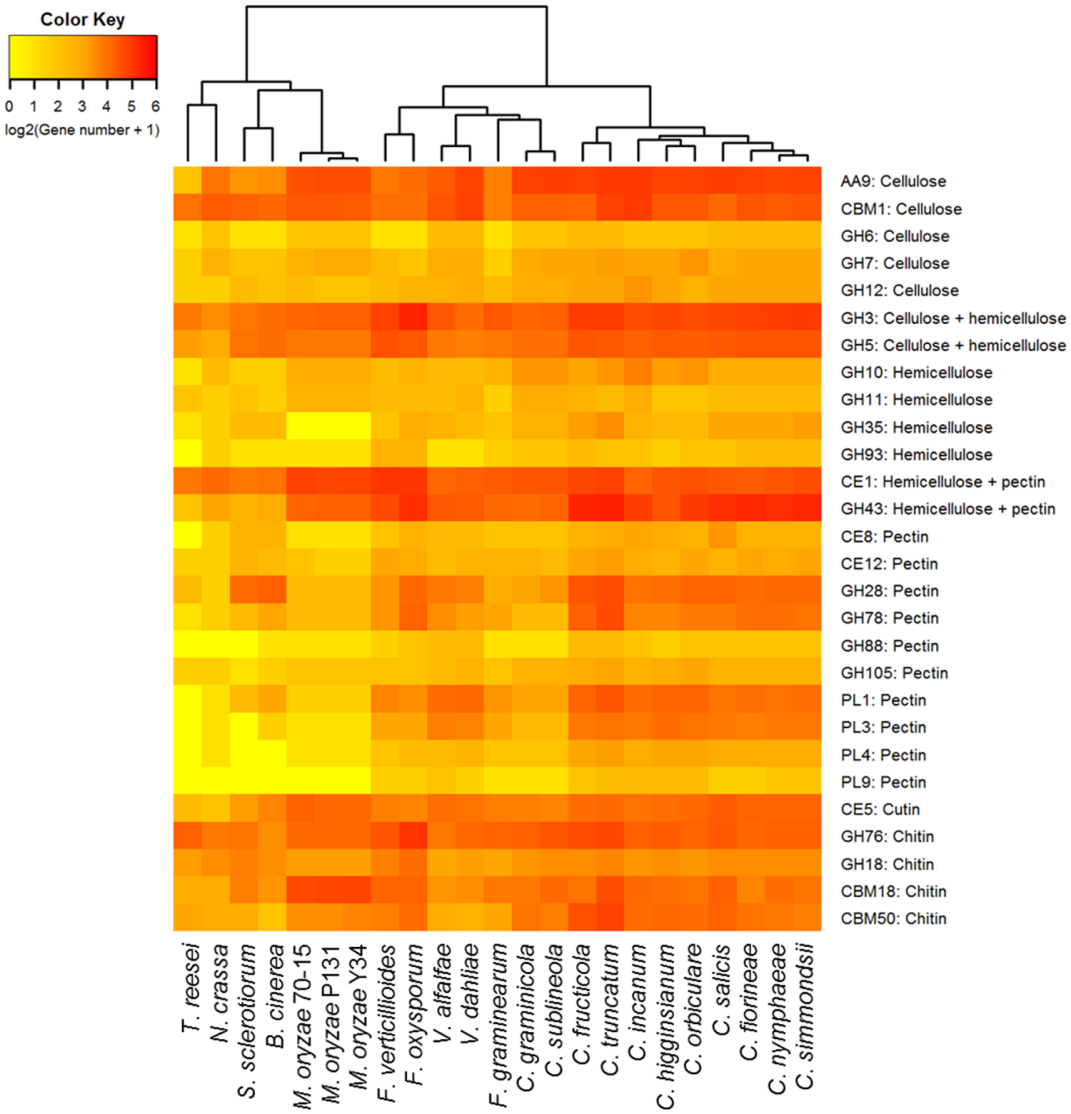
The heatmap showing important fungal and plant cell wall degrading CAZyme families in the proteomes of different fungi. The members of graminicola species complex that specifically infect monocot hosts clustered away from the rest of the *Colletotrichum* species while *C*. *fructicola* and *C*. *truncatum* with broad host range showed the maximum expansion of pectin degrading CAZyme families within the genus.

The heatmap of plant and fungal cell wall degrading enzymes (PCWDEs and FCWDEs) showed graminicola clade members, viz., *C*. *graminicola* and *C*. *sublineola* that specifically infect graminaceous monocot host, clustering away from the rest of the *Colletotrichum* species owing to the contraction of certain CAZyme families; while *C*. *fructicola* and *C*. *truncatum* with a broad host range formed a separate cluster due to the expansion of many families (Fig 5). 17 cutinases (CE5 family) were detected, 11 of which were homologous to *CtCut1*, a cutinase gene described previously for *C*. *truncatum* [86]. The chitin-binding domains belonging to the class CBM50 harboring 1–6 lysine motifs (*LysM*) and CBM18 were more abundant in *C*. *truncatum* genome as compared to other species. 7 genes had 3 CAZyme signatures; a chitin-binding CBM18 domain and up to three CBM50 domains as well as a GH18 catalytic domain with chitinase activity. 4 of these genes along with 10 other *LysM* genes (143–762 amino acid residues) were predicted to be secreted, while 3 were putative effectors (S9 Table). *CTRU_001009* had 9 orthologues that were specific to genus *Colletotrichum*, and *CTRU_002865* and *CTRU_002908* had homologues in other fungi, while 11 of these proteins were unique to *C*. *truncatum*.

The chitin- and cellulose-binding CBM1 family was specifically expanded in *C*. *incanum* (30) and *C*. *truncatum* (28) among the members of the *Colletotrichum* genus, with almost all genes predicted to be secreted. The genes containing CBM42 binding to arabinofuranose, were found in all fungi except *C*. *truncatum* and *C*. *incanum*. CBM67 binding to L-rhamnose showed high expansion specifically in *C*. *truncatum* (14) and *F*. *oxysporum* (15), whereas this family was absent in the members of graminicolous clade of *Colletotrichum* and also in the non-pathogenic fungi, *N*. *crassa* and *T*. *reesei*. Pectin-degrading enzymes (PL1 and PL3) showed the maximum expansion in *Colletotrichum* species when compared to other fungi. A pectin lyase family PL1 was observed in all fungi except *T*. *reesei*, and had highest expansion in *C*. *truncatum* (22).

### Proteases

**Fig 6:**
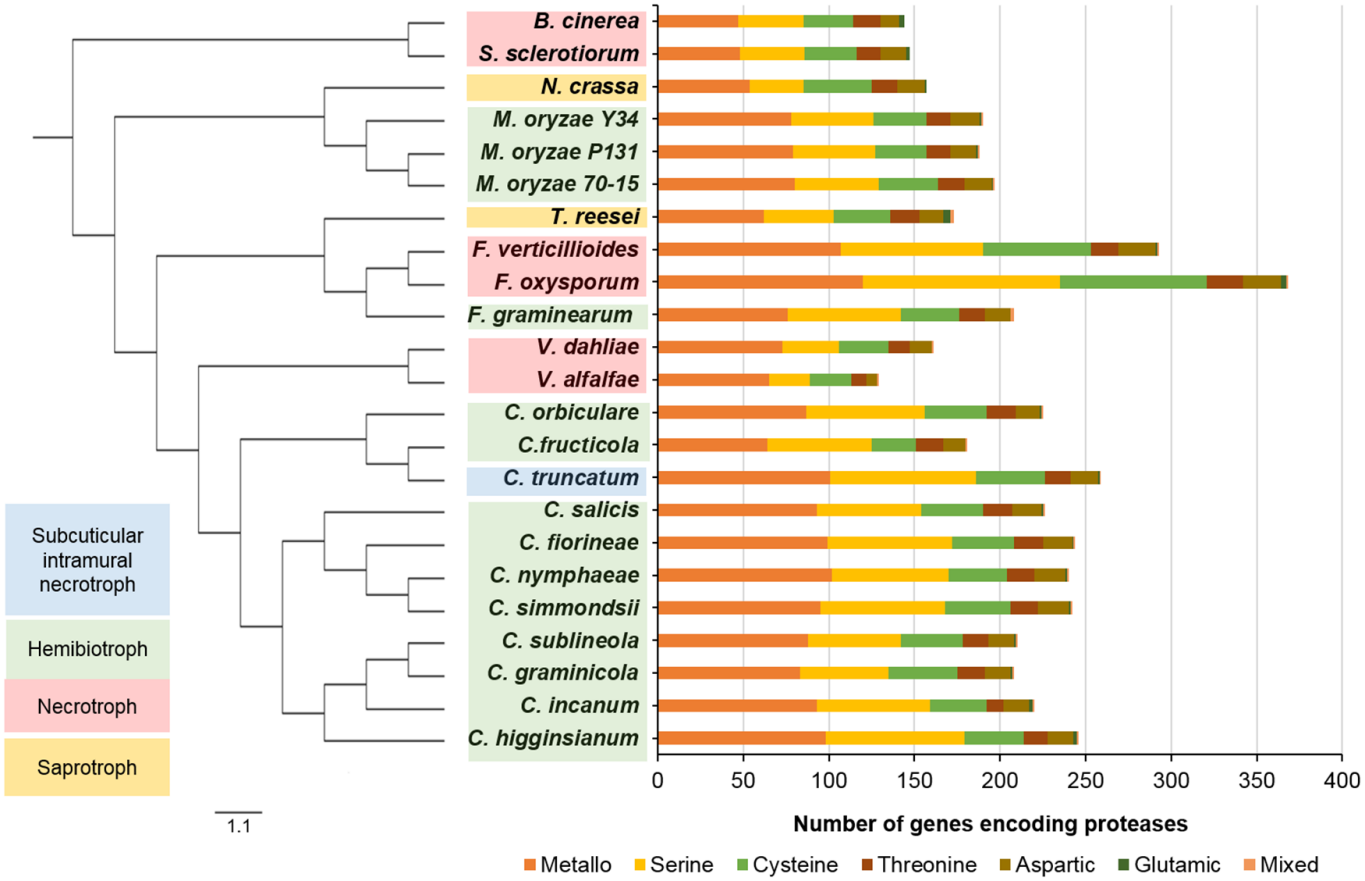
The comparative analysis of protease families in different fungal genomes. Phylogenetic tree of fungi with diverse lifestyles is shown with corresponding protease families in each fungal genome. *C*. *truncatum* had the largest protease component among *Colletotrichum* species due to expansion of metallo- and serine proteases.

Proteases complement the plant cell wall degrading enzymes and protect the fungi from host pathogenicity-related proteins released during fungal invasion. Batch BLAST search against MEROPS protease database [73] identified 258 genes in *C*. *truncatum* belonging to 76 families of proteases and 10 genes belonging to 4 protease inhibitor families. The genes from S09 family were screened since they were likely to be alpha/beta hydrolases (carboxylesterases and lipases, but not proteases). The metalloproteases (101) comprised the largest category of proteases in *C*. *truncatum*, followed by serine (85) and cysteine proteases (40), with similar trend in other fungi (Fig 6). *C*. *truncatum* had 71 secretory proteases that included 35 serine and 29 metalloproteases. 13 out of 20 subtilisins (S08A) were predicted to be secreted (S10 and S12 Tables). The two chitin-degrading, secreted fungalysins (M36) in *C*. *truncatum*, *CTRU_012332* and *CTRU_004392*, shared only 50% identity with each other and the former showed 91% identity to a *C*. *fructicola* fungalysin.

Interestingly, *C*. *fructicola* had the least number of proteases (185) among *Colletotrichum* species despite its wide host range. *C*. *graminicola* (213) and *C*. *subliniola* (215) also showed less diversity in proteases, similar to the trend observed with CAZymes (S11 Table). A total of 110 protease families were detected in all the fungi analysed, of which 46 families were common to all. The largest protease family in all the fungi studied was prolyl aminopeptidase (S33) followed by subtilisins (S08). The largest expansion of protease families was observed in *Fusarium* species, followed by *Colletotrichum* and *Magnaporthe* species. On the other hand, *V*. *alfalfae* (137) showed the least number of proteases among all the fungi. *T*. *reesei* (182) showed relatively large protease component than necrotrophic fungi like *Verticillium* spp, *Sclerotinia* and *Botrytis*; and non-pathogenic fungus *Neurospora*. The rare families included A11A that was observed only in *F*. *oxysporum* (7), *S*. *sclerotiorum* (6) and *C*. *incanum* (1).

### Secondary metabolite (SM) gene clusters

**Fig 7:**
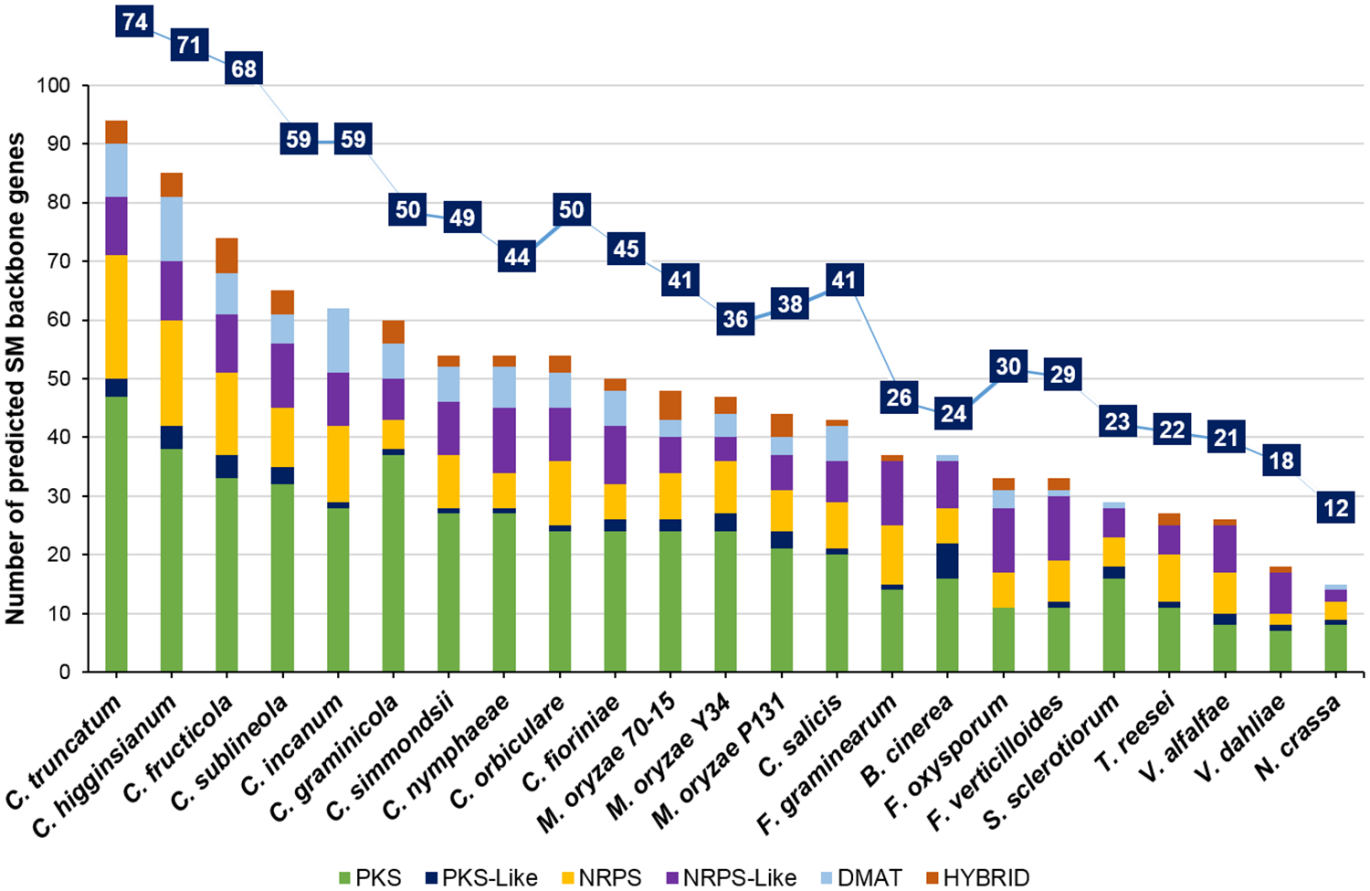
The comparative analysis of secondary metabolite (SM) gene clusters (line graph) and SM backbone genes (bar graph) predicted in different fungi using SMURF. *Colletotrichum* species had maximum expansion of SM backbone genes among all the fungi analysed, while *Verticillium* species showed contraction of these genes.

The SMs produced by the fungal phytopathogens are known to be associated with their pathogenicity and host range. The genes encoding the enzymes for the production of the SMs are located in the form of a cluster in the genome and their expression is transcriptionally co-regulated [87]. The comparative analysis of the SM gene clusters, predicted through SMURF, showed the highest number of such clusters in *Colletotrichum* species, ranging from 41 in *C*. *salicis* to 71 in *C*. *higginsianum* and 73 in *C*. *truncatum* (S13 Table), 2–4 fold higher than their close relatives in *Verticillium* (18–21 clusters) and *Fusarium* (26–30 clusters) (Fig 7). The SM backbone genes were mainly represented by 50 polyketide synthases (47 PKS and 3 PKS-like genes), 27 non-ribosomal protein kinases (17 NRPS and 10 NRPS-like genes), 9 dimethylallyl transferases (DMAT) and 4 PKS-NRPS hybrid genes in *C*. *truncatum*. The orthologue analysis identified 18 core SM backbone genes that were present in all the *Colletotrichum* species, including 6 PKS, 8 NRPS and 4 DMAT genes. The highest number of orthologues of SM backbone genes of *C*. *truncatum* was observed in *C*. *higginsianum* (59) and *C*. *fructicola* (58), respectively, most of which were PKS genes.

Another tool, antiSMASH predicted 73 clusters in *C*. *truncatum* genome, including 29 type1 PKS (T1PKS), 12 NRPS, 7 each of t1PKS-NRPS hybrids, terpene and indole type, 1 each of T3PKS-T1PKS, indole-T1PKS and indole-T1PKS-NRPS hybrid type of SM clusters along with 8 other clusters (S14 Table). Some of the genes in 9 of the 73 antiSMASH predicted SM clusters showed homology to the known clusters in other fungi. One of the T1PKS clusters (cluster 71) showed 100% similarity with an alternapyrone biosynthetic gene cluster of *Alternaria solani* (S5 Fig). 51–68 SM gene clusters were predicted in *C*. *scovillei*, *C*. *graminicola*, *C*. *fructicola* and *C*. *higginsianum*, which were much less than those predicted in a previous study [26].

### Cytochrome P450 monooxygenases (P450s) and transporters

P450s are the diverse, heme-thiolate proteins, which play an important role in primary and secondary metabolism, and fungal pathogenicity, and are often associated with the SM gene clusters [88]. 1,345 genes in *C*. *truncatum* had homologues in fungal cytochrome P450 database. 860 genes had more than 30% sequence identity to 347 proteins from the database with a minimum bit score of 60, showing a significant match. Comparative analysis of P450 proteins among all the fungi showed that many genes of *C*. *higginsianum* and *C*. *graminicola*, which were included in the database, were the core genes in the *Colletotrichum* genus (S15 Table).

Apart from P450s, transporters are also reported to be associated with the SM gene clusters and export of toxic SMs during host invasion and detoxification of fungal cells. 1,374 genes of *C*. *truncatum* had homologues (with >30% identity) in Transporter Classification Database (TCDB) with 340 genes belonging to the Major Facilitator Superfamily (MFS, 2.A.1), the largest category of secondary carriers involved in nutrient uptake and transport of toxins and drugs [89]. The second most expanded transporter family in *C*. *truncatum* was nuclear pore complex (NPC, 1.I.1) with 82 genes, involved in transport of mRNA and proteins across the nuclear envelope, followed by ABC transporter family (3.A.1) with 56 genes, which contains both uptake and efflux transport systems driven by ATP hydrolysis. The comparative analysis with other fungi showed the highest expansion of transporters in *F*. *oxysporum* and *F*. *graminicola* followed by *C*. *fructicola* and *C*. *truncatum* (S6 Fig). The electrochemical potential-driven transporters, which include a subclass of uniporters, symporters, antiporters (2.A) containing MFS (2.A.1), formed the largest class of transporters in all the fungi analysed.

### Putative pathogenicity genes

**Fig 8:**
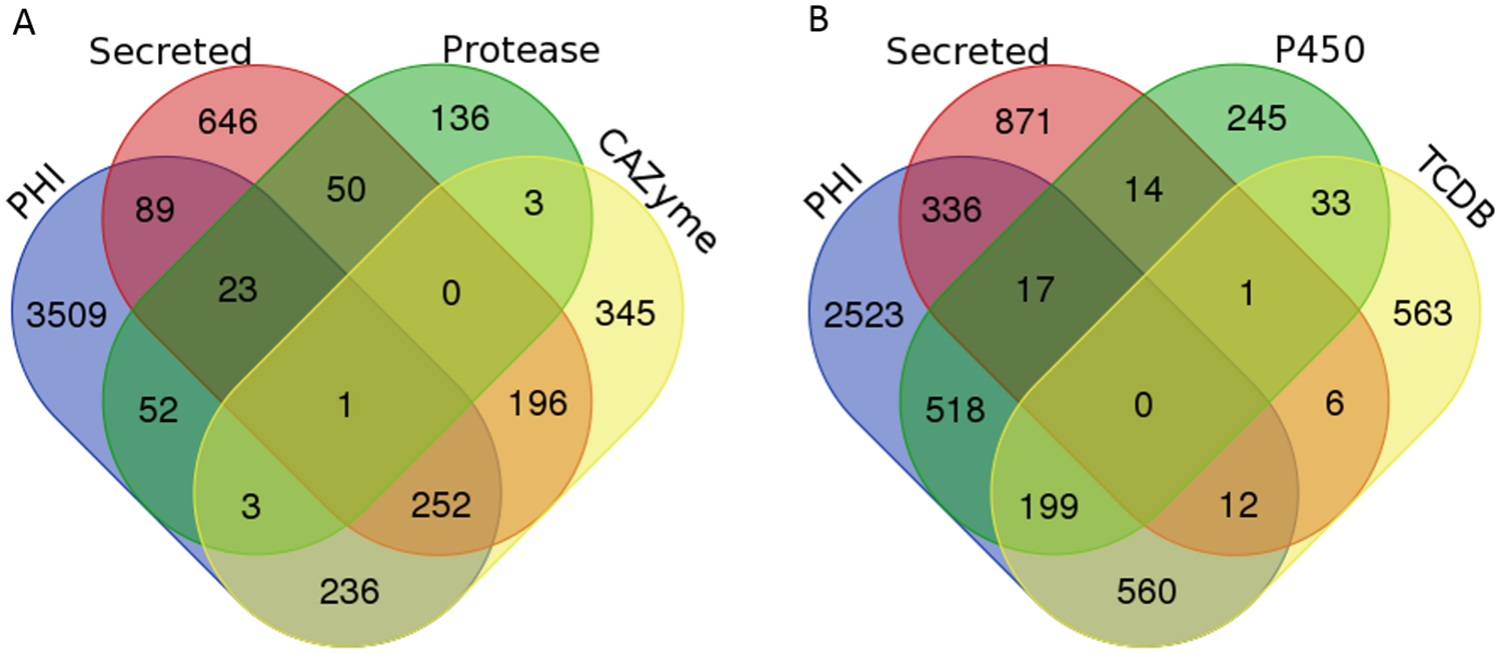
Venn diagram showing the overlap of different gene categories relevant to fungal pathogenicity. Overlap of secretory proteins and PHI-homologues with CAZymes and proteases (A), and cytochrome P450 and transporters with homologues in TCDB (B) representing the putative pathogenicity related genes with diverse functions.

Pathogen-Host Interactions database (PHI-base) comprises of the genes associated with pathogenicity that are experimentally validated in different pathogens [76]. A total of 4,165 genes with homologues in the PHI-base were identified in *C*. *truncatum*, many of which were important for the fungal pathogenicity and virulence (S16 Table). The majority of these genes belonged to the category that show no effect on pathogenicity on mutagenesis, while others belonged to different functional categories relevant for pathogenicity including secreted proteases, CAZymes, P450s and transporters (Fig 8). 15.7% (2,156) of the predicted *C*. *truncatum* genes had homologues in PHI-base which were reported to affect the host-pathogen interactions during infection. Out of 4,165 genes, 1,470 genes were associated with reduced virulence, 263 with loss of pathogenicity, 287 with mixed phenotype, 59 with hypervirulence and 38 with effector functions. The comparative analysis of PHI-base homologues from other fungi revealed the maximum number of homologous genes from all categories in *F*. *oxysporum*, *F*. *verticillioides*, *C*. *fructicola* and *C*. *truncatum*, while *M*. *oryzae* had the highest number of effectors (S7 Fig).

### Differential gene expression analysis

**Fig 9:**
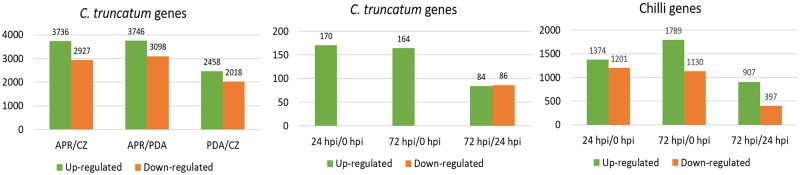
Differentially expressed genes under different conditions and infection stages *in vitro* and *in planta*. (A) Number of differentially regulated genes of *C*. *truncatum* in APR and PDA compared to CZ and APR compared to PDA. (B) Number of differentially regulated genes of *C*. *truncatum in planta* at 24 and 72 hpi compared to 0 hpi (control, uninfected chilli) and at 72 hpi in comparison to 24 hpi. (C) Number of differentially regulated genes of chilli for 24 and 72 hpi in comparison to 0 hpi.

The RNA-Seq data from the limited number of samples was undertaken to generate evidence for gene annotation and to get clues regarding differentially expressed genes among *in vitro* and *in planta* samples, generating pilot data before scaling up the experiments. The GFOLD (or generalized fold change) algorithm was employed for this purpose since it was specifically designed for single samples without biological replicates and gives stable and biologically meaningful rankings of differentially expressed genes [50]. The normalized gene expression levels in each sample were used to estimate the fold changes (log_2_ratio) and those with at least 2-fold change (log_2_fdc ≥1 and Gfold (0.01) > 1) were considered as differentially expressed (Fig 9).

65–88% of the *C*. *truncatum* genes were expressed at different stages of development (*in planta* or *in vitro*). A total of 8,442 genes were differentially regulated in APR (*in vitro* appresorial assay) and PDA (hyphae and conidia grown on solid medium) compared to CZ (hyphae grown in liquid culture), 746 of which were predicted to be secreted, including 146 putative effectors, 274 CAZymes and 46 proteases. The majority of genes up-regulated in APR and PDA were associated with oxidation-reduction processes, transmembrane transport, Zn ion binding, ATP binding and nuclear transport, and included MFS-1, heterokaryon incompatibility protein (HET), fungal transcriptional regulatory proteins with Zn(2)-Cys(6) binuclear cluster domains, etc. Analysis of *in planta* samples showed 174 up-regulated genes in 24 hpi and 72 hpi compared to 0 hpi, including 13 CAZymes. 61 common genes were up-regulated in all *in vitro* and *in planta* samples compared to their respective controls (S17 Table). The fungal genes up-regulated *in planta* at 24 and 72 hpi included protein kinases, MFS-1, ring finger proteins with a role in the ubiquitanation pathway (E3 ubiquitin-protein ligase). More than 2,500 chilli genes were differentially regulated in early infection stages (24 and 72 hpi) as compared to control (0 hpi).

## Discussion

The chilli anthracnose fungus, *C*. *truncatum* has been a serious threat for the major chilli producing countries for many decades. The wide host range and subcuticular intramural necrotrophic lifestyle make it an interesting candidate to study fungal pathogenicity and host specificity. With the development of tools for fungal transformation and availability of chilli genome sequences [36,90], chilli-*C*. *truncatum* pathosystem provides an excellent model to study host-pathogen interactions in which both the partners are amenable to genetic manipulation. The present study was undertaken with an aim to fill the lacunae in chilli-*Colletotrichum* interaction studies by providing a high quality reference genome sequence and annotation of this pathogen of a commercially important crop plant. The genome assembly of ~55 Mb was comparable to other sequenced *Colletotrichum* species except *C*. *orbiculare* (~90 Mb). The 6,793 contigs were assembled into 80 scaffolds, a low number for a genome sequenced on Illumina platform, due to the inclusion of two mate-pair libraries along with short insert paired-end reads for the assembly. This draft assembly represented the nuclear genome of *C*. *truncatum*, since no homology to the mitochondrial genome of other *Colletotrichum* species was observed.

Consensus gene models were predicted in *C*. *truncatum* through MAKER2 pipeline, taking into consideration the RNA-Seq data, homology with *Colletotrichum* species and SwissProt database as well as *ab initio* gene predictions from some of the best gene callers presently available for fungal genome annotation to enable accurate annotation of protein-coding genes. 13,724 genes were predicted with high confidence in *C*. *truncatum* as indicated by Annotation Edit Distance (AED) integrated in MAKER2. AED is a measure of the goodness of fit of an annotation to the evidence supporting it. AED of zero denotes perfect concordance with the available evidence (EST alignments, RNA-Seq and protein homology data), whereas a value of one indicates a complete absence of support for the annotated gene model [79]. In general, a well annotated genome is expected to have AED of less than 0.5 for 90% of the annotations and known domains for over 50% of its proteome [46]. >80% genes of the ‘gold-standard’ annotations of maize had AED scores of <0.2 [41]. In *C*. *truncatum*, 79% of the annotations had AED values of <0.2, which was very close to that of a well-annotated genome suggesting the reliability of these predictions. 74% of the total predicted proteins had a known IPR or Pfam domain and 66% had a homologue in SwissProt database. In contrast, only 2.6% genes had AED of 0.75–1, indicating little or no evidence support for these annotations. Interestingly, ~79% of the genes with AED of >0.75 had a Pfam domain, suggesting that they are not likely to be false positives. The other 21% genes with no known domain may include some unique genes and/or genes with undetectable expression or brief activation at certain stages, and can be prioritized for manual review. The *C*. *truncatum* genome assembly with small number of scaffolds, high N50 (1.6 Mb), detection of >99% of conserved eukaryotic/fungal genes, prediction of highly reliable gene models with less AED and presence of recognizable domains in a large number of predicted proteins point towards the high quality of the draft genome sequence of *C*. *truncatum* achieved in this study.

Synteny analysis of *C*. *truncatum* with *C*. *higginsianum* genome revealed a large fraction of genome which seems to be translocated or inverted in *C*. *truncatum*. Synteny analysis of *C*. *graminicola* with *C*. *higginsianum* and *C*. *fructicola* with *C*. *orbiculare* in earlier studies showed only 35–40% synteny [18,20], while >80% of the genes of the two species from graminicola species complex, *C*. *graminicola* and *C*. *sublineola* were syntenous [91]. Only 3% of the *C*. *truncatum* genome comprised of repetitive DNA elements (S2 and S3 Tables), which could be an under-estimation, likely due to the screening or collapse of the heterochromatin and other repetitive regions by the sequence assembly algorithm. The 4.75% of gaps observed in the assembly could also be attributed to the repetitive DNA, since some of the gaps that were inspected randomly had repetitive sequences flanking either the 5’ or 3’ ends. The completion of genome assembly and closure of the gaps in the existing sequence to get more contiguous assembly with fewer scaffolds will provide greater insights into the contribution of repetitive elements in the genome expansion and diversification of effectors. The predicted collinearity of existing scaffolds based on synteny mapping with *C*. *higginsianum* chromosomes can be used in future to get a nearly complete genome assembly.

The genome-wide comparative analyses of *C*. *truncatum* were carried out with 11 other *Colletotrichum* species (the proteomes for which are available in NCBI) and 8 other phylogenetically related fungi with diverse lifestyles including hemibiotrophs, necrotrophs and saprotrophs. The inferred phylogeny was consistent with the previous studies with *Colletotrichum* and *Verticillium* species diverging from *Fusarium*, pointing at their origin from a common ancestor. Hence, these genera are expected to share more homologues among each other and show similar expansion or contraction of certain gene families. ~89% of predicted genes had homologues in other *Colletotrichum* species reflecting the reliability of predicted gene models. Analysis of orthologues revealed the presence of 377 unique genes in *C*. *truncatum*, which had no homology to known proteins in SwissProt database and could be associated with its host-specific lifestyle. However, there is a possibility that predicted gene numbers may be inflated with incorrect or truncated gene structures which require manual curation.

The results of the comparative genomics were consistent with the previous findings that show the expansion of certain gene classes potentially involved in pathogenesis, in particular secreted proteins and effectors, CAZymes, proteases, SM synthesis and associated genes in *Colletotrichum* species when compared to other phytopathogenic fungi [18,20,25,92]. Though *Colletotrichum* genus is closely related to *Verticillium* species, the pathogenicity gene categories in all species including *C*. *truncatum* were similar to *Fusarium* and *Magnaporthe* species. *C*. *truncatum* had a diverse arsenal of such genes similar to the hemibiotrophic fungi when compared to necrotrophic and saptrotrophic fungi (except *Fusarium* species).

**Fig 10:**
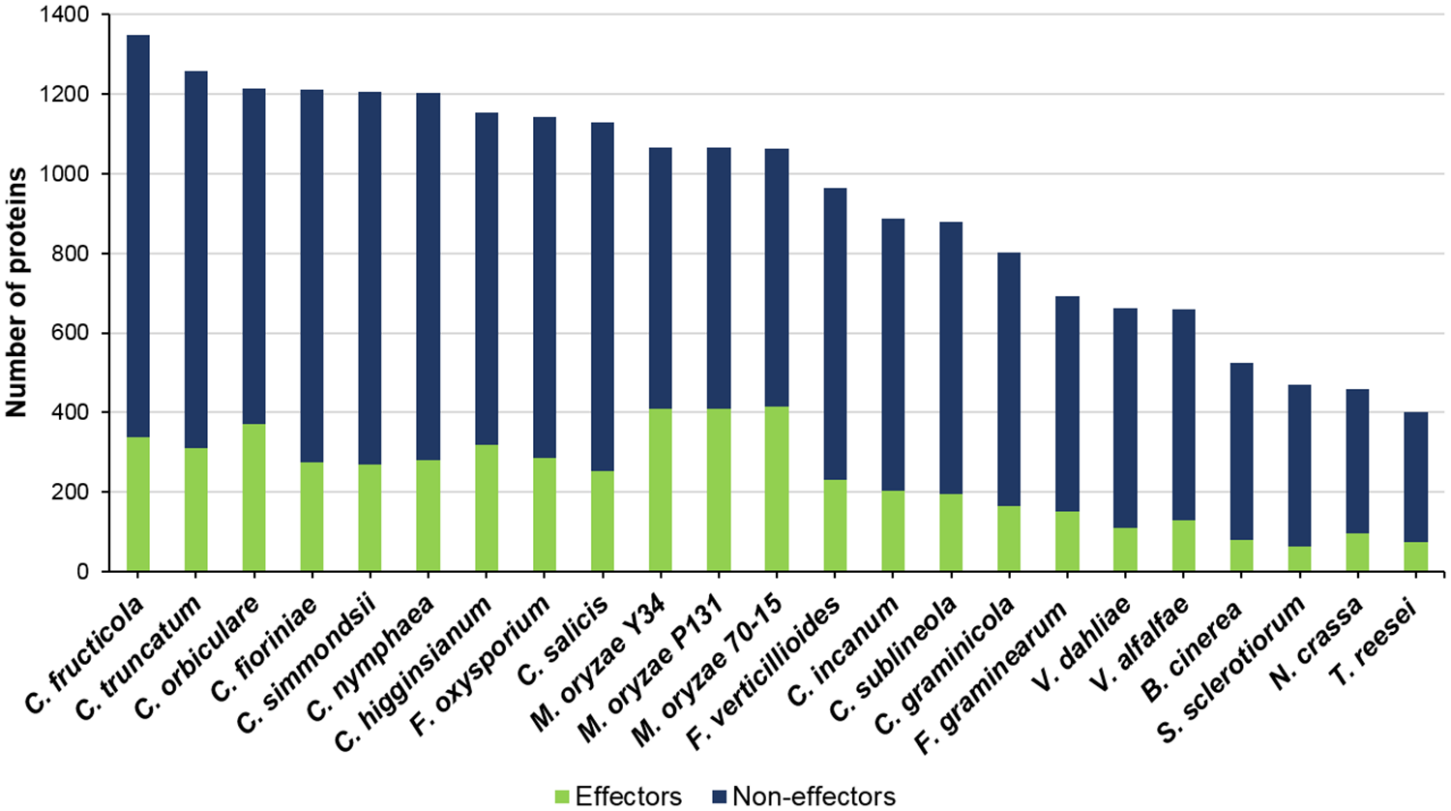
The number of secreted proteins and putative effectors, predicted using EffectorP, in all the fungal species analysed. *C*. *fructicola* and *C*. *truncatum* had the largest number of secreted proteins while *Magnaporthe* species had the largest effector component.

Pathogenic fungi overcome the host immune response by secreting effector molecules that manipulate cellular dynamics and help them evade the host defence responses and establish successful infection [93]. Effectors are typically lineage-specific, small, secreted, cysteine-rich proteins, most of which lack homology to known proteins, and have no recognizable domains [71,93]. However, some of the known effectors have homologues in related fungi as well as functional domains [94–96], though fungal effector proteins do not share a conserved host targeting signal like RxLR motifs of Oomycetes. A stringent strategy based on the tools described for fungal secretome prediction [81] was used in this study for the prediction of proteins that are highly likely to be secreted in *C*. *truncatum* as well as in other fungi. Though it resulted in reduction in the number of predicted secreted proteins reported in the previous studies through different tools (Fig 10), but reduced the probability of finding false positives in the secretomes of various fungi. However, use of such stringent strategies may result in some false negatives, similar reductions in the number of proteins in the refined secretomes of other fungi like *F*. *graminearum* [97], *S*. *sclerotiorum* [98] and *B*. *cinerea* [98] were observed using other stringent methods.

The *C*. *truncatum* secretome was rich in proteins like FAD oxidases, subtilisins, pectin lyases and putative effectors, which have been reported to play a role in host infection. The presence of the nuclear localization signal in many of the effectors implied their crucial role in the manipulation of host genes, probably related to defence, through translocation into the host nucleus and transcriptional reprogramming. 219 candidate effectors were shortlisted based on their lack of homology to known proteins or domains. 36 species-specific proteins with no known domains and no homologues in the SwissProt database were predicted to be secreted in *C*. *truncatum*, 25 of which were putative effectors and were expressed *in planta* and/or *in vitro*. The homologues of some of the known fungal effectors that are present in all pathogenic fungi like LysM, NLPs etc. were identified in the *C*. *truncatum* genome as well. The homologues of *C*. *higginsianum* extracellular LysM protein effectors (*ChELPs*) [99] that were shown to be essential for appresorial function and suppressing chitin-triggered plant immunity, and another effector that is secreted from appresorial pore during early penetration (*ChEC6*) [82] were observed in *C*. *truncatum* as well and possibly might play an important role during host infection. Interestingly, *CtNudix*, a nudix hydrolase effector expressed during transition to necrotrophy in the cowpea anthracnose pathogen *C*. *lentis* [100], had no homologue in *C*. *truncatum* which might be in tune with its host specific necrotrophic lifestyle which contrasts hemibiotrophy. It was reported to have homologues only in hemibiotrophic fungi, but not in biotrophic or necrotrophic fungi [92,101].

Analysis of the major polysaccharide degrading enzyme (CAZyme) families with a wide range of substrates revealed lineage specific expansion of pectin degrading CAZymes (GH28, GH78, PL1 and PL3), cutinases (CE5) in *C*. *truncatum* and *C*. *fructicola*, both of which cause fruit rots in a number of hosts. These CAZyme families probably aid in the degradation of thick cuticle and complex cell walls of fruits rich in pectins and provide an evolutionary advantage to these fruit rot fungi. On the other hand, contraction of these families along with AA class in *C*. *graminicola* and *C*. *sublineola* points towards their dispensability for monocot infecting graminicolous clade as reported earlier [26]. Some *Colletotrichum* species like the members of gloeosporioides and acutatum clade, employ a stealth strategy, wherein they were reported to be either physiologically inactive on fruits until the ripening process starts, thus enabling them to avoid the host defence mechanisms within unripe, pre-climacteric fruits, or by endophytic species colonizing the living host tissues asymptomatically [102]. The abundance of CBM50 (LysM effectors) along with CBM18, the other chitin-binding module mainly associated with CE4 chitin deacetylase, and GH18 chitinase (apart from CBM50), and chitin- and cellulose-binding CBM1 family was a prominent feature of *C*. *truncatum* CAZyme and secretome components. These secreted CAZymes may play an important role during early infection by reducing the release of chitooligosaccharides by action of plant chitinases or sequestering the released chitin oligomers that act as Microbe Associated Molecular Patterns (MAMPs) and thereby preventing their recognition by plant immune system [20,64,94,99]. The expression of these CAZymes after host invasion may be responsible for the stealth strategy employed by *C*. *truncatum* during endophytic or quiescent phase of infection that has been observed even in a susceptible host like chilli [16,103].

Apart from CAZymes, proteases are the major plant cell wall degrading enzymes, of which serine- and metallo-proteases are the most important classes present in pathogenic fungi including *Colletotrichum* species. The aspartic protease family was also expanded in the *Colletotrichum* species that encounter acidic environment while infecting fruits. Interestingly, *Trichoderma* had larger protease component than non-pathogenic fungi like *Neurospora* and some of the necrotrophs. The expansion of proteases and CAZymes for lignocellulosic substrates in *Trichoderma* may be attributed to its saprophytic lifestyle that involves the digestion of dead plant material and cell wall components. *C*. *truncatum* showed a variety of metalloproteases, which possibly reflects a link between certain metalloproteases and necrotrophic lifestyle. The two fungalysins in *C*. *truncatum* (*CTRU_004392* and *CTRU_012332*), the evolutionarily conserved chitin degrading enzymes present in all fungi analysed, had characteristic features of zinc metalloproteases, viz; a fungalysin/thermolysin propeptide (FTP) domain and M36 peptidase domain with catalytic activity and HEXXH motif [104]. Most of the fungi in family Sordariomycetes contain a single fungalysin gene. In a previous study, the phylogeny based on fungalysins from different fungi suggested that a gene duplication event probably might have occurred prior to the appearance of the Sordariomycetes, followed by numerous gene loss events of one or two copies in most of the members of Sordariomycetes [104]. This may explain the presence of two copies of this gene in *C*. *truncatum* and *Verticillium* species.

Subtilisin (S08A) was the most expanded family of alkaline proteases in *C*. *truncatum* as well as other species of the genus. Local alkalinization of the host tissue due to fungal secretion of ammonia was reported in many *Colletotrichum* species which provides an optimum pH to secreted hydrolases such as pectin-degrading enzymes [18,20]. The expansion of acidic and alkaline proteases in *Colletotrichum* species gives these fungi the ability to withstand a wide pH range encountered at different stages of infection in a number of hosts. There were reports of horizontal gene transfer (HGT) of subtilisin genes from plants to *Colletotrichum* fungi and hence such genes are named as *Colletotrichum* plant-like subtilisins (CPLS) [105]. *C*. *truncatum* had 4 proteins homologous to CPLSs that were similar to subtilisin-like protease (SBT1.7) of *Arabidopsis thaliana* based on SwissProt database search. *CTRU_000179* and *CTRU_000180* were found to be truncated and hence were fused and named as *CTRU_000179A*. All three CPLSs (*CTRU_000179A*, *CTRU_007447* and *CTRU_008341*) had different sequences from each other, but retained the three characteristic domains, viz., peptidase inhibitor I9 domain (PF05922), the PA domain (PF02225) and the peptidase S8 domain (PF00082) with conserved catalytic triad at active site. When these three proteins were subjected to BLASTP analysis against the NCBI non-redundant database, among the top hits were subtilisin-like proteases from plants, including many *Brassica* species, *Gossypium* species, lotus, tobacco etc. and the proteins from two other distantly related ascomycetes, *Rhynchosporium* and *Diaporthe* species. This finding further supported the theory of HGT of some of the subtilisin genes from plants into an ancestor of *Colletotrichum* species. Apart from the subtilisins, there were 268 more proteins in *C*. *truncatum* proteome that had their best hits in *A*. *thaliana* in SwissProt database, while many proteins had hits in other plants like rice, maize, tobacco, tomato, chilli, cucurbits, etc. Additionally, many genes appeared to be of bacterial, rather than fungal, origin. This finding suggests that with additional analyses, an evidence for the HGT in many of these genes could be obtained in the future.

During host invasion, the fungal pathogens produce secondary metabolites that affect their pathogenicity and virulence, while many host plants produce antimicrobial compounds, SMs and toxins. Fungi counteract these toxins by enzymatic degradation or export them through membrane transporters. The comparative genomic analysis showed a genus-specific expansion of SM gene clusters in *Colletotrichum* species, including P450 genes, transporters and TFs suggesting their transcriptional co-regulation that may affect their virulence and help in nutrient uptake and export of toxins and antimicrobial compounds produced by the host. The expansion in SM gene clusters predicted through SMURF in *C*. *truncatum* (73), *C*. *higginsianum* (71) and *C*. *fructicola* (68) may also reflect their broad host range. An expansion of PKS and NRPS backbone genes, including some unique genes in *C*. *truncatum*, shows greater capacity of production of these SMs, which may play a significant role in host invasion by enabling appresorial penetration and pathogenicity. Another tool used for SM gene cluster prediction, antiSMASH predicted 73 clusters in *C*. *truncatum*, some of which had homologous genes from known SM clusters of other fungi (S5 Fig). >80 clusters were predicted for other *Colletotrichum* species in a previous study using an earlier version of antiSMASH (v1.2.2) [26]. The reduction in number of predicted clusters for some of these fungi (<68) could be attributed to the advanced version of the online tool (fungiSMASH) and the parameters used for the analysis.

The Pathogen-Host Interactions database (PHI-base) serves as a multi-species catalogue of experimentally validated pathogenicity genes (through gene/transcript disruption experiments) from plant and animal pathogens (bacteria, fungi, protists and nematodes) that are manually curated from peer-reviewed publications [76,106]. 15% of *C*. *truncatum* proteome had homologues in PHI-base associated with fungal pathogenicity including effectors, CAZymes, proteases, transporters and P450 genes. Most of the *C*. *truncatum* genes had homology to genes from *F*. *oxysporum* and *M*. *oryzae* due to a plethora of research carried out on these model fungi making them the highest represented pathogens in PHI-base. The majority of these genes was associated with reduced virulence. These genes, along with the genes associated with loss of pathogenicity and homologues of characterized effectors could be good candidates for further research. The comparative analysis of PHI-base homologues revealed a rich repertoire of pathogenicity genes in *C*. *truncatum* and *C*. *fructicola* among all fungi except the *Fusarium* species.

The RNA-Seq data was primarily used to support the annotation of protein coding genes and to get a glimpse of the differential gene expression at various developmental stages. GFOLD, which is one of the best available algorithms for differential expression analysis from single samples without biological replicates, was used to analyse RNA-Seq data. More fungal genes were expressed *in vitro* than *in planta* probably due to lesser representation of the fungal partner in the latter. *In vitro* appresoria (APR) showed the highest number of differentially regulated genes compared to the mycelial and conidial biomass grown in rich medium (PDA) and mycelia grown in minimal medium (CZ). Many secreted CAZymes, proteases, TFs and PHI-base homologues were up-regulated in appresoria as well as *in planta*, which may be needed to penetrate the host cell wall during initial infection. 80% of the species-specific genes in *C*. *truncatum* were expressed *in vitro* and/or *in planta*, some of which had at least one of the IPR or Pfam annotations indicating that these represent the genuine genes and not artifacts. Though the *in vitro* samples included about half of the total predicted genes that showed differential expression, the *in planta* samples had only a small fraction of such fungal genes, possibly due to the infection assay adopted, which avoided wounding the chilli surface that might have resulted in less representation of fungal transcripts. Even though the differential gene expression studies were performed without the biological replicates, the genes expressed during appresorial development and initial infection of chilli provide the candidates for future studies to understand the molecular mechanism of fungal pathogenicity.

Deciphering the genetic basis of the infection mechanism and host-specific lifestyle of fungal pathogens is crucial for a better understanding of host-fungal interactions and to develop effective and novel strategies to combat the pathogen. With the availability of whole genome sequences of chilli [90][36] and its major pathogens, *C*. *scovillei* (deposited as *C*. *acutatum*) [24] and *C*. *truncatum* (present study) in the public domain, the chilli-*Colletotrichum* pathosystem provides an attractive model to study host-pathogen interactions. Due to the unavailability of annotated genes of *C*. *scovillei* in public domain, direct comparison of pathogenicity genes among these two species, showing necrotrophy on the same host, was not possible. However, 26 genes of *C*. *truncatum*, which have homologs only in *C*. *scovillei*, could be candidates for further studies to delineate the host-specific infection strategy of both the fungi. The existence of conserved fungal genes and core genes from the genus *Colletotrichum* in *C*. *truncatum* genome reflects the good quality of assembly and gene predictions that were achieved in the present study. RNA-Seq analysis not only provided evidence for the gene structure prediction, but also provided the preliminary cues to design high throughput experiments to study gene regulation and a list of species-specific candidate genes for experimental validation and functional characterization. The comparative genomic analyses of the gene categories relevant for fungal pathogenicity revealed the conserved genes among *Colletotrichum* species with a broad host range and unique genes, including effectors; SM associated genes, PHI homologues, expanded CAZymes and proteases, and the absence of hemibiotrophic effector *CtNudix* in *C*. *truncatum*. Many of the genes belonging to these pathogenicity relevant classes could be associated with its host specific subcuticular intramural necrotrophic lifestyle and need to be functionally characterized. Summarily, this study provides a high quality reference genome sequence and annotation of genes with putative roles in pathogenicity and host interaction of an important *Colletotrichum* species, which would serve as a genomic resource to facilitate further functional and evolutionary studies of this agronomically important fungal pathogen and to develop novel disease control measures.

## Supporting information

S1 Fig The sizes of genomes and GC-content of different ascomycetes fungi and their corresponding proteomes. *C*. *truncatum* genome and proteome sizes were comparable to other *Colletotrichum* species, except *C*. *orbiculare* and *F*. *oxysporum* which had the largest genome and proteome among the fungi analysed, respectively.
(TIF)

S2 Fig The phylogenetic tree of *Colletotrichum* species obtained from neighbor joining (NJ) analysis based on multilocus alignment of 5 genes (ITS, CHS-1, HIS3, ACT and TUB2) using 1000 bootstrap replicates. *Monilochaetes infuscans* was taken as an outgroup. The species with genome sequence available at the time of sequencing of *C*. *truncatum* (MTCC no. 3414) are marked with red solid circles. Bootstrap support values (1000 replicates) above 50% are shown at the nodes.
(TIF)

S3 Fig The distribution of number (>3, blue circles) and percentage (>3%, orange circles) of cysteines in cysteine-rich secreted proteins along with their corresponding protein sizes. The small (below 300 amino acids), secreted, cysteine-rich proteins can be considered as candidate effectors if they lack homology to known proteins and functional domains.
(TIF)

S4 Fig The distribution of sizes of proteins (Y-axis) in secretome of different fungi analysed (X-axis). The median sizes of all secreted proteins in *Colletotrichum* species were below 300 amino acids (aa), except for *M*. *oryzae* strains which had median at 200 aa and encode maximum number of reported effectors among all the fungi analysed. The sizes of all the putative effectors in *C*. *truncatum* were below 358 aa except for *CTRU_010949* (508 aa). The outliers were removed from the plot.
(TIF)

S5 Fig The homology of alternapyrone biosynthetic gene cluster of *Alternaria solani* in *C*. *truncatum* secondary metabolite gene cluster 71 of type 1 polyketide synthase (T1PKS). The corresponding homologous genes are shown in red, green and blue colours. MFS: Major Facilitator Superfamily (transporter).
(TIF)

S6 Fig The comparison of different classes of transporters among all the fungi analysed. *Fusarium* species had exceptionally high number of transporters among all fungi followed by *Colletotrichum* species. The electrochemical potential-driven transporters, which include a subclass of uniporters, symporters, antiporters (2.A) containing Major Facilitator Superfamily (2.A.1), formed the largest class of transporters in all the fungi analysed, followed by uptake and efflux transport systems driven by ATP hydrolysis, containing ABC transporter family (3.A.1).
(TIF)

S7 Fig The number of genes with functionally characterized homologues in manually curated Pathogen-Host Interaction database (PHI-base) in all the fungi analysed. (A) The homologues from some of the most abundant categories of genes in PHI-base. The category of genes with unaffected pathogenicity phenotype (validated through mutagenesis) were the largest among PHI-base homologues in all fungi followed by the category of genes with reduced pathogenicity. (B) The homologues from some of the least represented categories of genes in PHI-base. The category of genes with hypervirulence phenotype was the largest among all fungi except *M*. *oryzae* that had the largest effector component. *F*. *oxysporum* and *F*. *verticillioides* had the maximum number of PHI-base homologues reflecting the amount of experimental data available for this genus.
(TIF)

S1 Table Summary statistics of the pre-processed reads from the RNA samples of *C*. *truncatum*. (XLSX)

S2 Table The repetitive elements in *C*. *truncatum* genome predicted using RepeatMasker through homology-based approach using all fungal sequences from RepBase Update 20140131. (XLSX)

S3 Table The repetitive elements in *C*. *truncatum* genome predicted using RepeatMasker through de novo repeats identified in the genome by RepeatModeler. (XLSX)

S4 Table The details of predicted genes of *C*. *truncatum* and gene categories relevant to pathogenicity. (XLSX)

S5 Table The synteny analysis between *C*. *higginsianum* (1) and *C*. *truncatum* (2). (XLSX)

S6 Table Homologues of some of the known effectors in *C*. *truncatum*. (XLSX)

S7 Table Species-specific genes in *C*. *truncatum* with no homologues in other *Colletotrichum* species and the SwissProt database. (XLSX)

S8 Table The comparison of carbohydrate-active enzymes (CAZymes) in *C*. *truncatum* with *Colletotrichum* species and other fungi. (XLSX)

S9 Table The secretory LysM proteins with corresponding numbers of CBM 50, CBM18 and GH 18 domains. (XLSX)

S10 Table The most abundant and important proteases and protease inhibitors in the proteome and secretome of *C*. *truncatum*. (XLSX)

S11 Table The comparison of protease families and protease inhibitors in *C*. *truncatum* proteome with *Colletotrichum* species and other fungi. (XLSX)

S12 Table The comparison of secreted protease families and protease inhibitors in *C*. *truncatum* with *Colletotrichum* species and other fungi. (XLSX)

S13 Table The secondary metabolite gene clusters in *C*. *truncatum* predicted by SMURF. (XLSX)

S14 Table Secondary metabolite gene clusters of *C*. *truncatum* predicted by online antiSMASH (fungiSMASH version 4.0.0rc1). (XLSX)

S15 Table The comparison of genes of different fungi with top BLASTP hits in cytochrome P450 database. (XLSX)

S16 Table The BLASTP analysis of homologues of *C*. *truncatum* genes in Pathogen-Host Interaction database (PHI-base). (XLSX)

S17 Table Fold changes in *C*. *truncatum* genes common for *in vitro* samples (APR and PDA in comparison CZ) and *in planta* samples (24 and 72 hpi in comparison with 0 hpi). (XLSX)
