# Supplementary figures and images for: Genome sequencing and comparative genomics reveal a repertoire of putative pathogenicity genes in chilli anthracnose fungus Colletotrichum truncatum
Source: PLoS One. 2017 Aug 28;12(8):e0183567. doi: 10.1371/journal.pone.0183567 (PMC5573122; doi:10.1371/journal.pone.0183567)

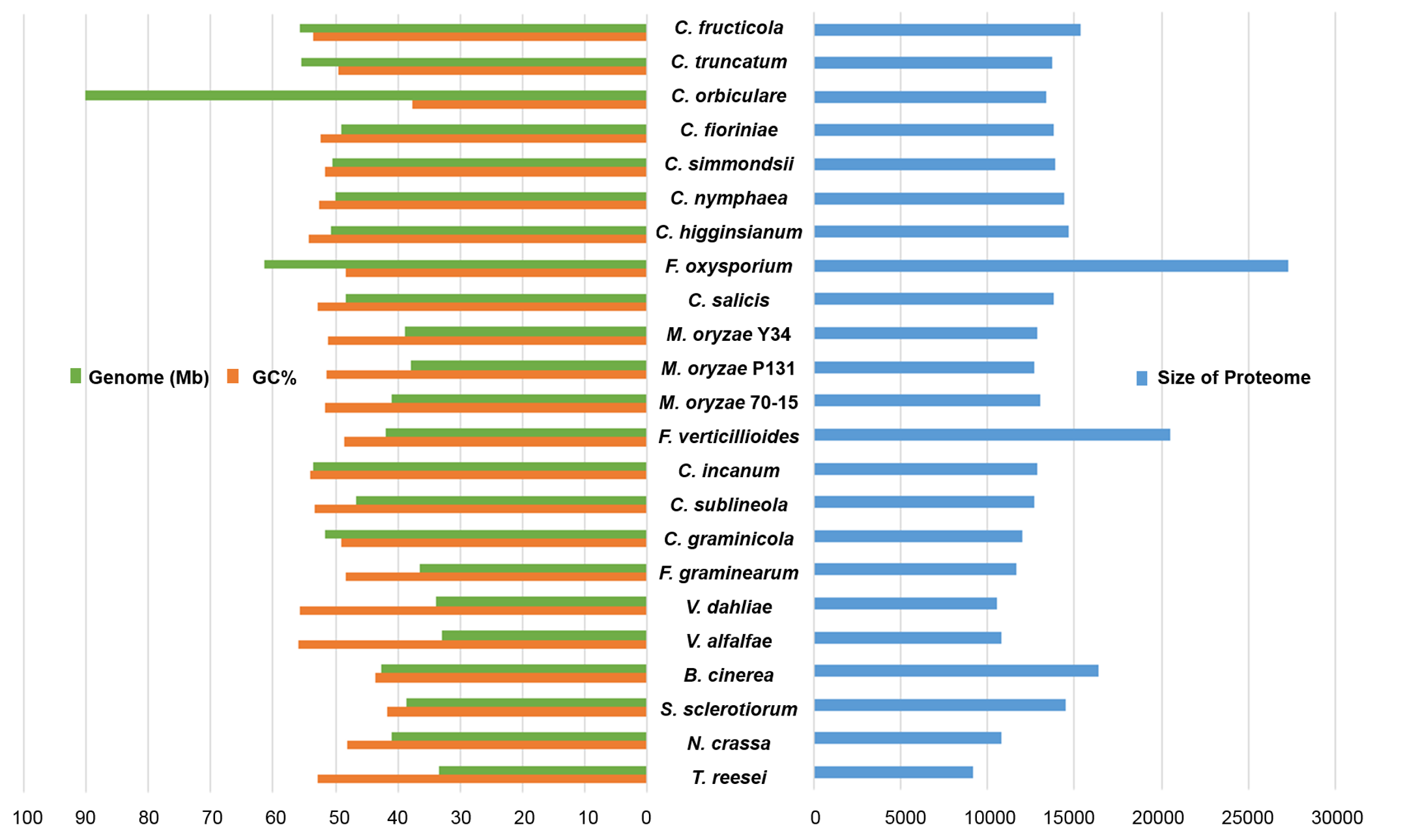

Supplement: S1 Fig — C. truncatum genome and proteome sizes were comparable to other Colletotrichum species, except C. orbiculare and F. oxysporum which had the largest genome and proteome among the fungi analysed, respectively. (TIF) [file pone.0183567.s001.tif]

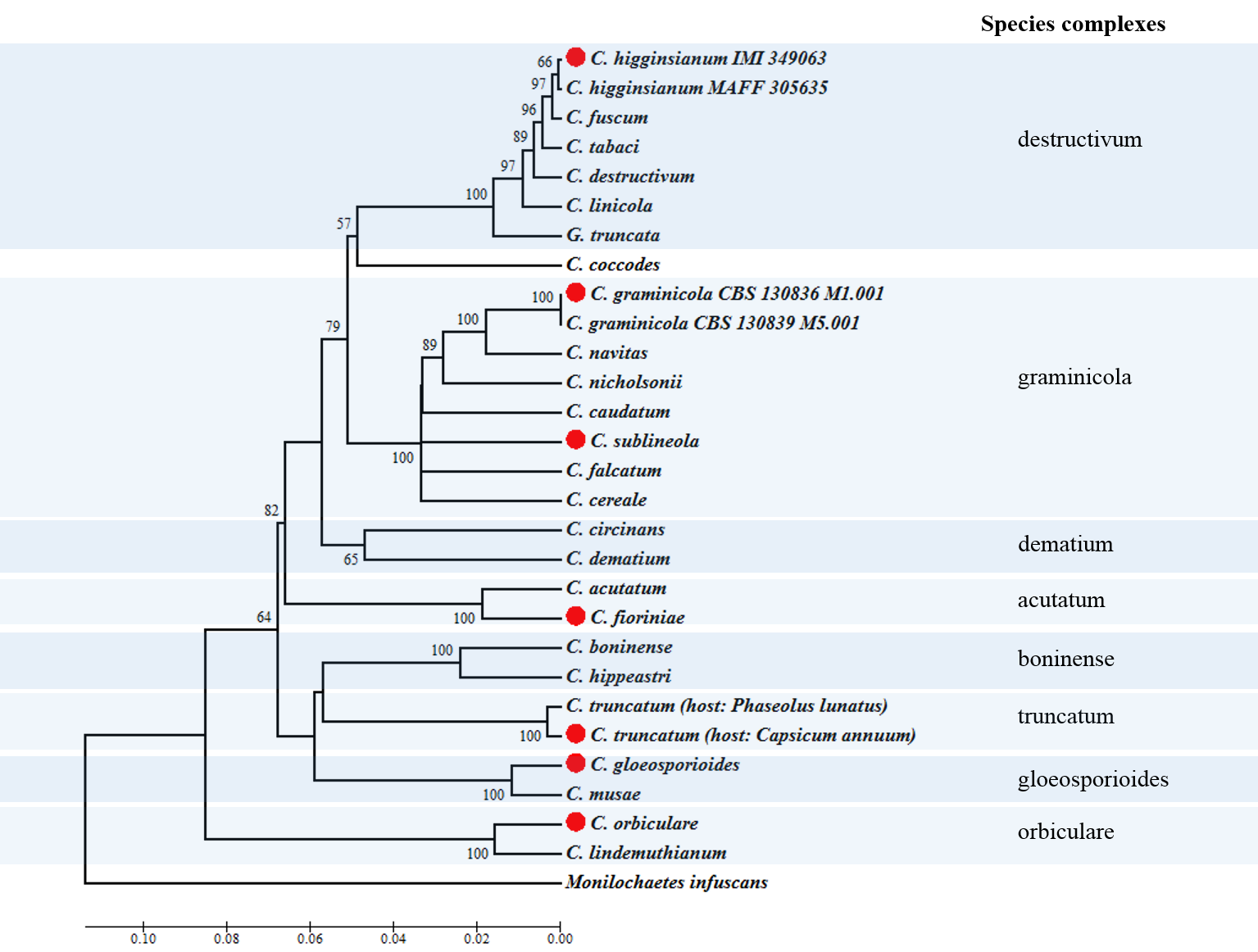

Supplement: S2 Fig — Monilochaetes infuscans was taken as an outgroup. The species with genome sequence available at the time of sequencing of C. truncatum (MTCC no. 3414) are marked with red solid circles. Bootstrap support values (1000 replicates) above 50% are shown at the nodes. (TIF) [file pone.0183567.s002.tif]

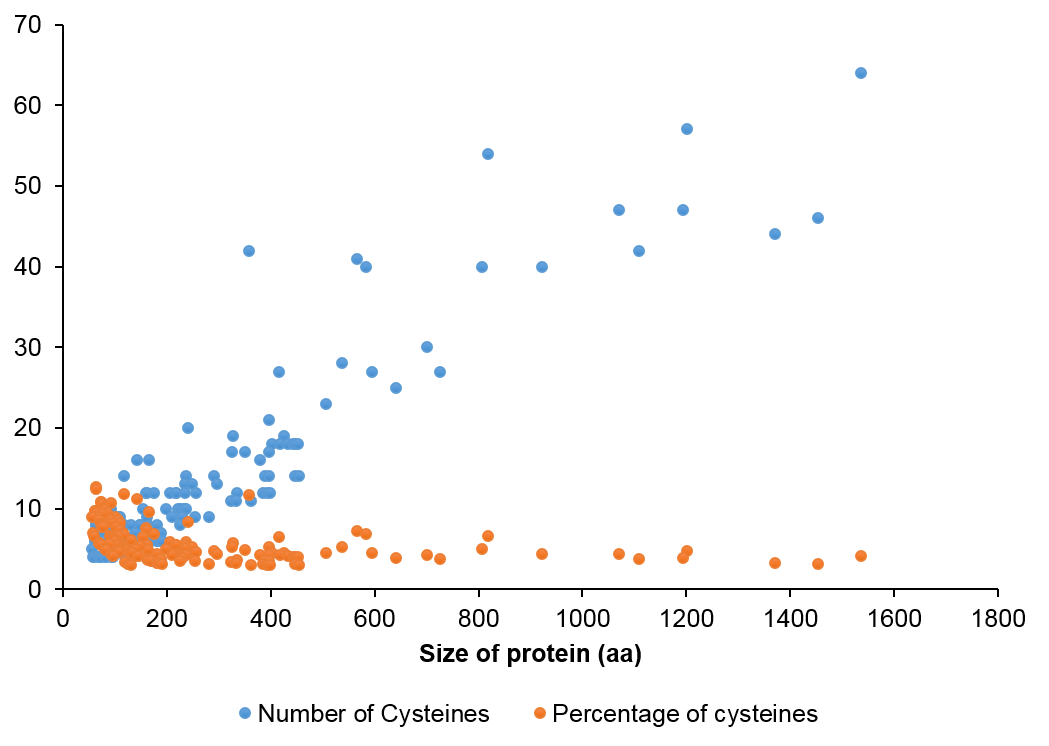

Supplement: S3 Fig — The small (below 300 amino acids), secreted, cysteine-rich proteins can be considered as candidate effectors if they lack homology to known proteins and functional domains. (TIF) [file pone.0183567.s003.tif]

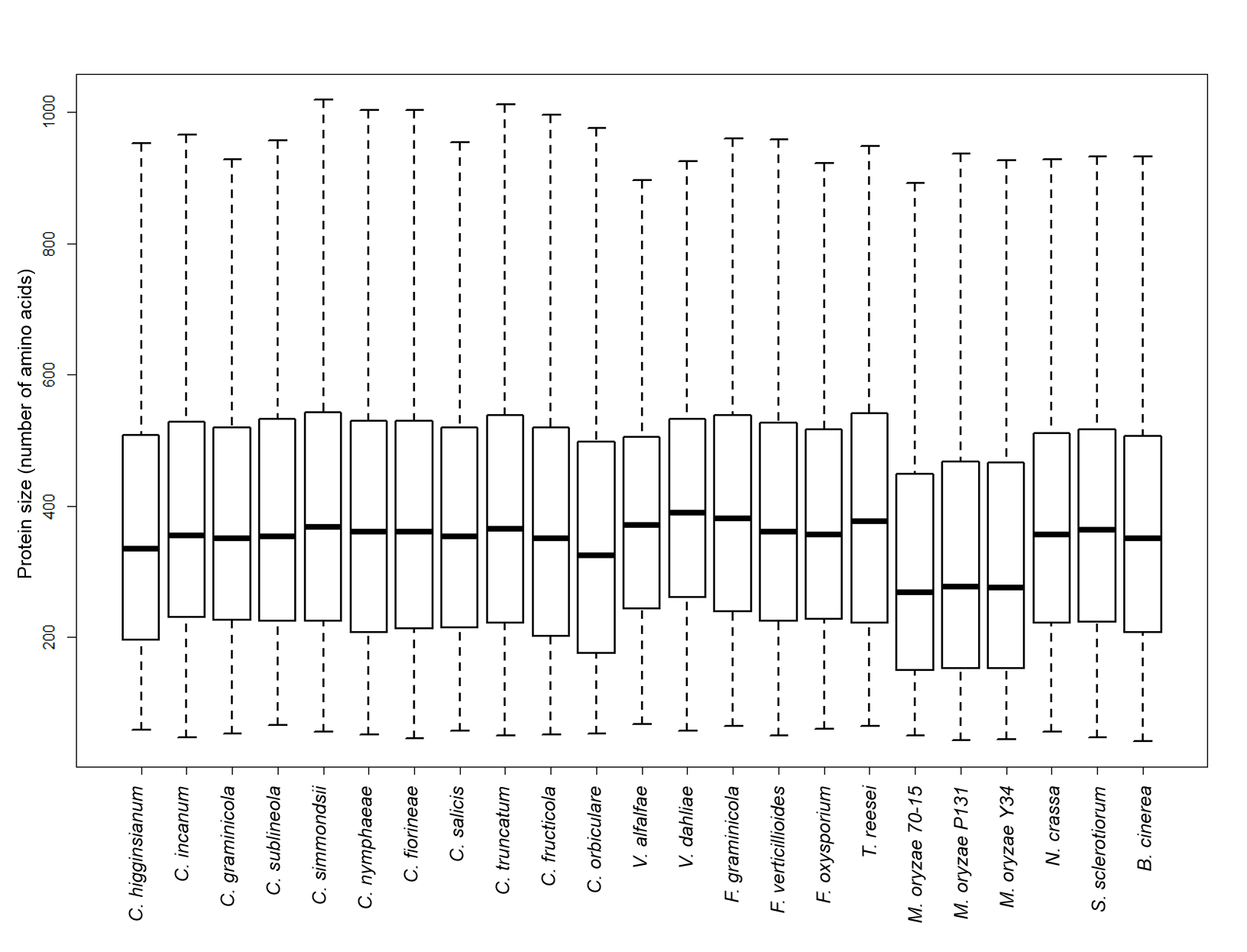

Supplement: S4 Fig — The median sizes of all secreted proteins in Colletotrichum species were below 300 amino acids (aa), except for M. oryzae strains which had median at 200 aa and encode maximum number of reported effectors among all the fungi analysed. The sizes of all the putative effectors in C. truncatum were below 358 aa except for CTRU_010949 (508 aa). The outliers were removed from the plot. (TIF) [file pone.0183567.s004.tif]

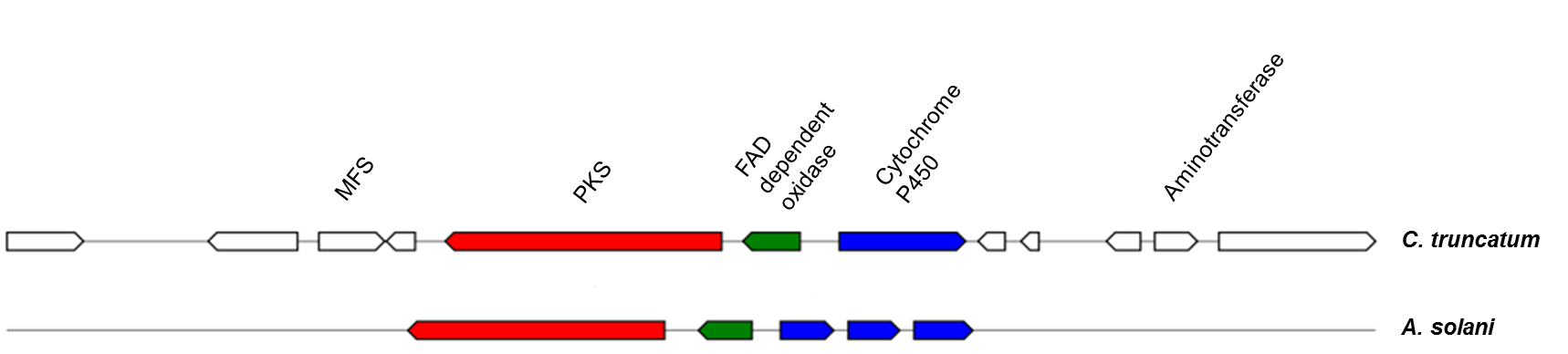

Supplement: S5 Fig — The corresponding homologous genes are shown in red, green and blue colours. MFS: Major Facilitator Superfamily (transporter). (TIF) [file pone.0183567.s005.tif]

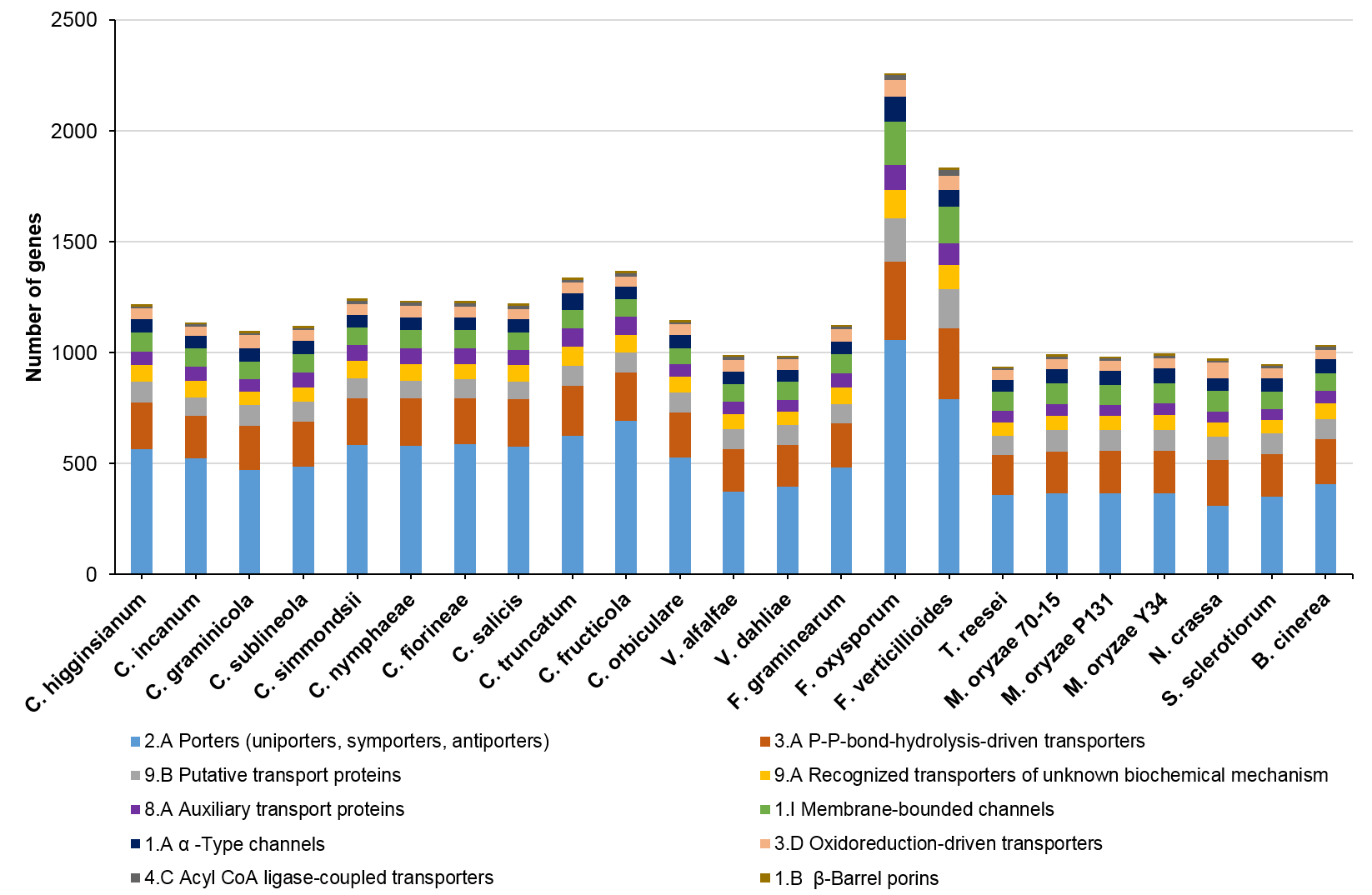

Supplement: S6 Fig — Fusarium species had exceptionally high number of transporters among all fungi followed by Colletotrichum species. The electrochemical potential-driven transporters, which include a subclass of uniporters, symporters, antiporters (2.A) containing Major Facilitator Superfamily (2.A.1), formed the largest class of transporters in all the fungi analysed, followed by uptake and efflux transport systems driven by ATP hydrolysis, containing ABC transporter family (3.A.1). (TIF) [file pone.0183567.s006.tif]

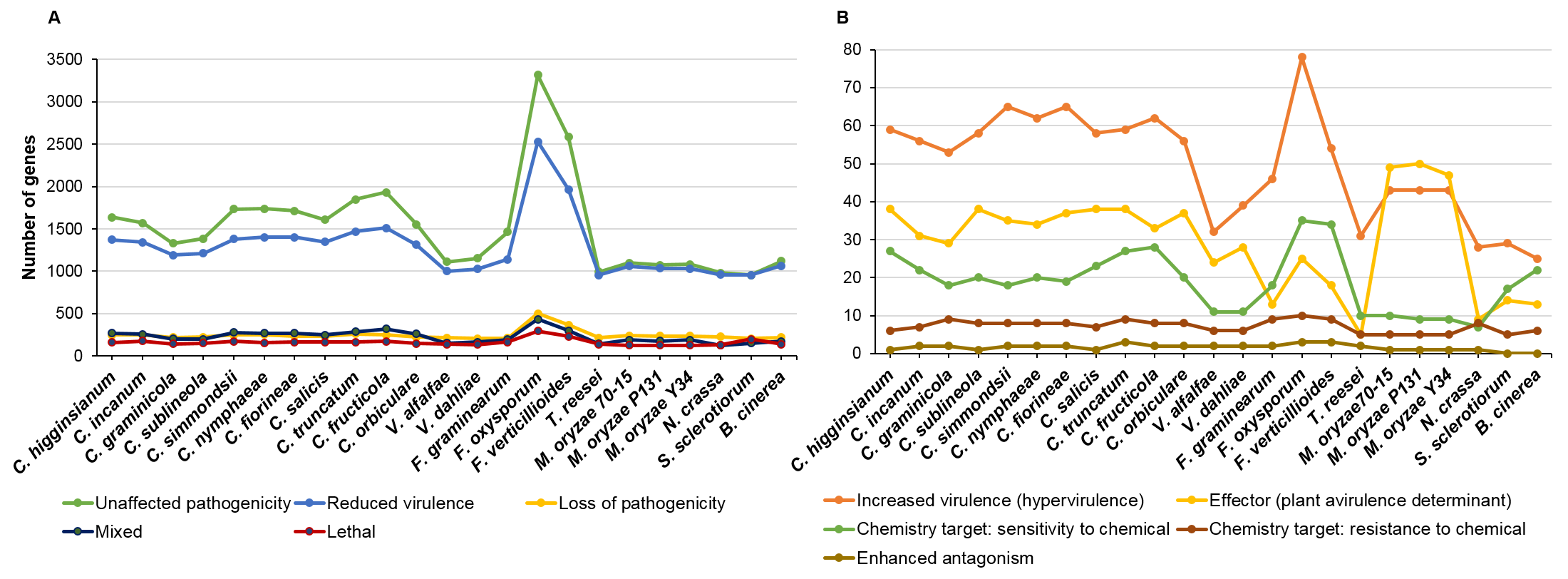

Supplement: S7 Fig — (A) The homologues from some of the most abundant categories of genes in PHI-base. The category of genes with unaffected pathogenicity phenotype (validated through mutagenesis) were the largest among PHI-base homologues in all fungi followed by the category of genes with reduced pathogenicity. (B) The homologues from some of the least represented categories of genes in PHI-base. The category of genes with hypervirulence phenotype was the largest among all fungi except M. oryzae that had the largest effector component. F. oxysporum and F. verticillioides had the maximum number of PHI-base homologues reflecting the amount of experimental data available for this genus. (TIF) [file pone.0183567.s007.tif]
